# Strategies for the implementation of palliative care education and organizational interventions in long-term care facilities: A scoping review

**DOI:** 10.1177/0269216319893635

**Published:** 2020-02-03

**Authors:** Danni Collingridge Moore, Sheila Payne, Lieve Van den Block, Julie Ling, Katherine Froggatt

**Affiliations:** 1 International Observatory on End of Life Care, Lancaster University, Lancaster, UK; 2 VUB-UGhent End of Life Care Research Group, Vrije Universiteit Brussel (VUB), Brussels, Belgium; 3 European Association for Palliative Care, Vilvoorde, Belgium

**Keywords:** Long-term care facilities, care homes, nursing homes, palliative care, end-of-life care, palliative medicine, scoping review, literature review, implementation, intervention

## Abstract

**Background:**

The number of older people dying in long-term care facilities is increasing; however, care at the end of life can be suboptimal. Interventions to improve palliative care delivery within these settings have been shown to be effective in improving care, but little is known about their implementation.

**Aim:**

The aim of this study was to describe the nature of implementation strategies and to identify facilitators and/or barriers to implementing palliative care interventions in long-term care facilities.

**Design:**

Scoping review with a thematic synthesis, following the ENTREQ guidelines.

**Data sources:**

Published literature was identified from electronic databases, including MEDLINE, EMBASE, PsycINFO and CINAHL. Controlled, non-controlled and qualitative studies and evaluations of interventions to improve palliative care in long-term care facilities were included. Studies that met the inclusion criteria were sourced and data extracted on the study characteristics, the implementation of the intervention, and facilitators and/or barriers to implementation.

**Results:**

The review identified 8902 abstracts, from which 61 studies were included in the review. A matrix of implementation was developed with four implementation strategies (facilitation, education/training, internal engagement and external engagement) and three implementation stages (conditions to introduce the intervention, embedding the intervention within day-to-day practice and sustaining ongoing change).

**Conclusion:**

Incorporating an implementation strategy into the development and delivery of an intervention is integral in embedding change in practice. The review has shown that the four implementation strategies identified varied considerably across interventions; however, similar facilitators and barriers were encountered across the studies identified. Further research is needed to understand the extent to which different implementation strategies can facilitate the uptake of palliative care interventions in long-term care facilities.

**What is already known about the topic?**

- The provision and quality of palliative care delivered in long-term care facilities (LTCFs) varies and does not always meet the needs of the residents.
- Interventions to improve palliative care have been shown to lead to improvements in the quality of care received by long-term care facilities residents.
- The implementation of such interventions and the factors that facilitate their uptake within an long-term care facilities are not well understood.

**What this paper adds?**

- This paper provides a scoping review of implementation strategies used by palliative care interventions in long-term care facilities.
- This review has identified four organizational strategies for the implementation of palliative care interventions: facilitation, education/training, internal engagement and external engagement.
- Three developmental stages comprise the implementation process: conditions to introduce the intervention, embedding the intervention within day-to-day practice and sustaining ongoing change.

**Implications for practice, theory or policy**

- The implementation strategies used varied across the studies identified; how implementation can support intervention uptake requires further investigation.
- The implementation strategies used to implement palliative care interventions in long-term care facilities are underreported, and separating characteristics of an intervention from the implementation process is complex. Further guidance is needed on the reporting of implementation strategies.
- The findings of this review may inform the development and implementation of future palliative care interventions in this setting and how they can be implemented more effectively.

## Background

Long-term care facilities (LTCFs) are increasingly becoming the final place of care for older adults. Across the globe, long-term care facilities are a common place of death for older adults,^1^ especially among those with dementia.^2^ Using the definition provided by Froggatt and Reitinger,^3^ a long-term care facilities is a collective institutional setting where care is provided for older people who live there for an undefined period of time, 24 h/day, 7 days/week. The care provided includes on-site provision of personal assistance with activities of daily living; nursing and medical care may be provided either on-site or from nursing and medical professionals external to the setting.^3^

Despite death being a natural progression as an individual ages, providing palliative care in long-term care facilities is complex. The majority of long-term care facilities residents live with more than one chronic condition, and dementia or high levels of cognitive impairment are common. Knowing when a resident is dying can be hard to predict, as residents with multiple chronic, life-limiting conditions may experience periods of both decline and improvement in their health before death.^4^ In Europe, long-term care facilities are generally staffed by registered nurses and care assistants; staff turnover can be high and pay relatively low, with limited opportunities for further education, on the job training or professional development. Staff members often have limited knowledge of the palliative care needs of older adults, especially in terms of managing pain and other symptoms at end of life.^5^

As defined by the World Health Organization,^6^ palliative care refers to

‘an approach that improves the quality of life of patients and their families facing the problem associated with life-threatening illness, through the prevention and relief of suffering by means of early identification and impeccable assessment and treatment of pain and other problems, physical, psychosocial and spiritual’.

To deliver high-quality care at end of life, long-term care facilities require a specific approach to palliative care that is appropriate to both the needs of the residents being cared for and the staff members working within the facilities.

The European Association for Palliative Care Taskforce on Palliative Care in Long-term Care Settings for Older People mapped approaches to developing and delivering palliative care between countries using a modified typology of change at international, national, regional and organizational levels.^3,7^ At an organizational level, initiatives to ensure long-term care facilities residents received palliative care could be through providing designated units (i.e. palliative care beds), care based (i.e. symptom management), care planning based (i.e. advance care planning), and organizational multicomponent interventions (i.e. Gold Standards Framework for Care Homes) or education and training.^7^ Interventions at an organizational level to improve the delivery of palliative care in long-term care facilities have demonstrated improvements, including increasing the numbers of completed advance directives,^8^ reducing deaths outside the long-term care facilities,^9^ improving end-of-life communication between families and clinicians^10–12^ and increasing staff knowledge and confidence.^13–15^

The implementation of these interventions, and the factors that facilitate their implementation, is less well understood. It is unclear how different approaches to implementation may affect the uptake of an intervention, and there is little consensus on how interventions can be embedded and sustained within an increasingly complex setting. Despite the urgent need to improve palliative care within long-term care facilities, identifying optimum ways of implementing palliative care has yet to be addressed.

## Aim and objectives

This scoping review explores the implementation strategies used in organizational-level interventions that aim to improve palliative care in long-term care facilities. It aims to identify the implementation strategies used to support palliative care interventions in long-term care facilities and the facilitators and/or barriers to implementation. This study is, to our knowledge, the first to attempt to explore the implementation process supporting the introduction of palliative care interventions in long-term care facilities.

### Design

This scoping review was designed and conducted using guidance from Arksey and O’Malley.^16^ As the focus is the process of implementation rather than outcomes, a scoping review allows the mapping of how the intervention was implemented rather than only the effectiveness of the strategies used. The scoping review method follows a five-step process: identifying the research question, identifying relevant studies, study selection, charting the data and collating, summarizing and reporting the results.^16^

## Stage 1: identifying the research question

The first stage of the scoping review was to identify the primary research question of the review by clarifying what was considered to be important. Two review questions were identified:

1. What implementation strategies were used to support the delivery of palliative care interventions in long-term care facilities?
2. What are the facilitators and/or barriers to successful implementation?

The review was restricted to studies published from 2007 onwards, which marked the publication of the first national End-of-Life Care Strategy, globally, for England and Wales.^17^ It was limited to studies published in English.

## Stage 2: identifying relevant studies

The systematic search strategy for the review was developed in line with guidance published by the Cochrane Handbook for Systematic Reviews of Interventions.^18^ The search strategy included a combination of free text terms and subject indexing terms, such as MeSH and Emtree. The search strategy was developed through the identification of key terms in the title and abstract of relevant studies already known to the research team.

The following electronic databases were searched for articles published in peer-reviewed journals: MEDLINE, EMBASE, PsycINFO, Cumulative Index to Nursing and Allied Health Literature (CINAHL), Proquest, the Cochrane Library, including the Cochrane Methodology Register, Cochrane Central Register of Controlled Trials (CENTRAL), Cochrane Database of Systematic Reviews (CDSR), Database of Abstracts of Reviews of Effect (DARE), Health Technology Assessment (HTA) database and NHS Economic Evaluation Database (NHS EED), Web of Science, the Campbell Library, SCOPUS and Social Care Online. The sample strategy used for MEDLINE in this research is shown in the Supplementary Material. In addition, papers were identified through reviewing the reference lists of publications which met the inclusion criteria and study protocols identified in the search. Reverse citation searches were also undertaken on papers which were included using the ISI Web of Science Citation Databases. Grey literature was excluded as our interest was on research-based publications. Databases were searched in September 2018.

## Stage 3: study selection

**Figure 1.**
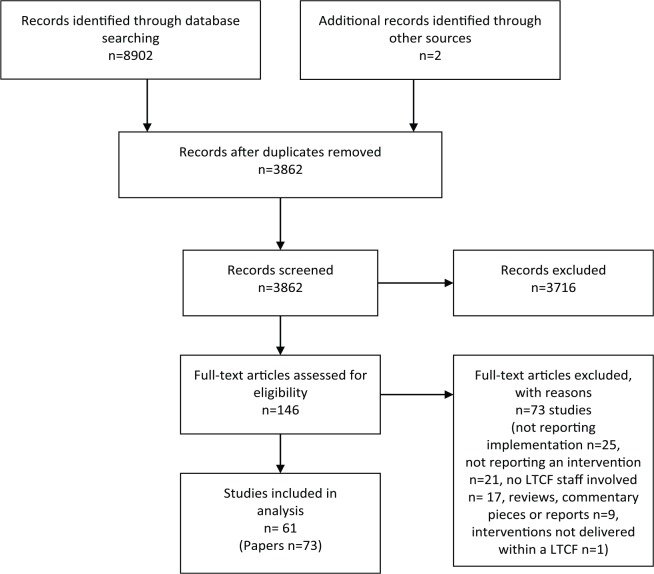
PRISMA flowchart.

The process of study selection is shown in the PRISMA flowchart in Figure 1. Inclusion criteria for the initial (title and abstract) screening were developed through discussion with the research team and were piloted by two researchers on a sample of 100 randomly selected papers. Title and abstract reviewing was applied by one researcher (D.C.M.), with a final decision made by a senior researcher (K.F.) where required. The inclusion criteria were modified and refined based on the findings. The review included studies that discussed delivery strategies for, or any information on facilitators and/or barriers to, implementing palliative care interventions for older adults living in long-term care facilities. The full paper review was conducted by two researchers (D.C.M. and A.H.) independently and a decision about whether each paper met the inclusion criteria was made. References for excluded studies and the reason for their exclusion were recorded.

## Stage 4: charting the data

Data from each study were extracted independently by two researchers and organized in Excel on four categories: the study design, the intervention, the implementation of the intervention and facilitators and/or barriers to implementation. Information regarding the study design and the intervention was extracted initially, allowing for further information on implementation to be contextualized. Data were extracted on the author and year, country, study design, long-term care facilities type and number of long-term care facilities in the study, duration of the intervention, description of the intervention, the main outcome measures or methods used and an overview of the study findings. In cases where two papers reported on the same study, quantitative and qualitative outcomes were reported separately.

Data on implementation of the interventions were extracted and mapped regarding facilitation, education/training and internal and external engagement, as defined in Table 1. Finally, data about facilitators and/or barriers to implementation of the intervention were extracted. This was drawn from findings sections, including data extracts and the author’s discussion of the findings of the intervention. Quotes from the papers and page numbers were extracted and tabulated.

**Table 1.** Data extracted on implementation and categorization criteria.

Theme	Definition
Facilitation	Facilitation referred to whether the intervention was facilitated, and if so, whether the facilitation was internal or external, the training or expertise of the facilitator and the contribution of the facilitator. Internal facilitation was defined as facilitation provided by a staff member employed within the LTCF and external facilitation was defined as a person external to the LTCF facilitating the intervention.
Training or education	Training referred to whether there was an education element to the intervention, and if so, how it was delivered and to whom.
Internal engagement	Internal engagement referred to whose behaviour the intervention was aiming to change to improve palliative care within the LTCF, that is, care home staff, managers and unregulated care providers.
External engagement	External engagement referred to whether or not any aspect of the intervention involved joint working, that is, between specialist palliative care services, primary care or hospitals. Data on joint working were only extracted where there was specific discussion of the intervention incorporating joint working, as opposed to embedding the intervention in current practice.

LTCF: long-term care facilities.

## Stage 5: collating, summarizing and reporting the results

The review methodology used was based on the guidance for selecting methods for qualitative evidence synthesis.^19^ The review team applied the RETREAT criteria, which informs the choice of qualitative synthesis method used based on the aims and characteristics of the review. The review question, the epistemology underpinning the review, time frame, resources, team expertise, audience and type of data being synthesized were discussed and thematic synthesis identified as an appropriate approach.^20^

Any discussion of facilitators and/or barriers to the implementation of an intervention was extracted verbatim from the included papers as quotes. The quotes were read and coded line by line using free codes to develop a code bank. These codes were used to develop descriptive themes and reorganized into hierarchical groups for discussion within the research team. In the final stage, analytical themes were generated and fed into a cyclical process whereby themes were generated and applied to the grouped codes. The ENTREQ statement and the PRISMA Extension for Scoping Reviews (PRISMA-ScR) were used to guide the reporting of the approach used for qualitative data synthesis.^21,22^

## Results

The searches of the electronic databases identified 8902 abstracts, based on the inclusion criteria detailed in Table 2. After removal of duplicates and studies not meeting the inclusion criteria, 146 abstracts were identified as potentially relevant studies. A further 73 papers were excluded on reviewing full papers. Two additional studies were identified through reverse citation and reference list searches. A total of 73 papers were included in the review, which reported on 61 studies; the characteristics of these studies are detailed in the Supplementary Material. Two studies reported three interventions; therefore, 65 interventions are reported. Of the included studies, 39% (*n* = 24) were conducted in the United Kingdom, 26% (*n* = 16) in the United States of America and Canada, 18% (*n* = 11) in the rest of Europe, 15% (*n* = 9) in Australia and New Zealand and 2% (*n* = 1) in China. Study design varied and were described by the publication as an implementation/evaluation study (52%, *n* = 32), quasi-experimental design or pre-test/post-test (28%, *n* = 17), randomized controlled trial (10%, *n* = 6), qualitative study (5%, *n* = 3) and feasibility or pilot study (5%, *n* = 3).

**Table 2.** Inclusion and exclusion criteria.

	Inclusion criteria	Exclusion criteria
Participants	The review focused on strategies for the implementation of palliative care interventions for older adults living in LTCFs. Older adults were defined as adults aged 65 years or above; in studies where only group descriptive statistics are reported, care facilities where average age of the group was aged 65 years or above were included.	Studies which looked at other places of residence where care is provided, which do not meet the definition of an LTCF, were excluded from the study. This included hospitals, sheltered housing or residential housing with home care services. In addition, facilities, such as hospices, which specifically care for residents approaching end of life, were excluded from the study.
Outcomes	The primary outcome of interest was how the intervention was implemented. This could include delivery strategies or any information on facilitators and/or barriers to implementing interventions.	None
Study design	All studies were included if they implemented an intervention, either through quantitative or qualitative methods. Evaluation, implementation or pilot studies were included.	Protocol papers were excluded; however, the study was followed up to see if potential outcome papers had subsequently been published.
Intervention	The review included research studies which provided information or discussed the implementation of organizational level interventions that aim to improve the provision or delivery of palliative care in LTCFs. The broad areas for interventions included • providing designated units, • care based (i.e. symptom management), • care planning (i.e. advance care planning), • organizational multicomponent interventions (i.e. Gold Standards Framework for Care Homes) and • education or training.	Studies were excluded if they discussed the development of a palliative care intervention without any information about the implementation process, or only reported attitudes towards the facilitators and/or barriers to delivering palliative care in general.

LTCF: long-term care facilities.

In terms of setting, 51% were reported as based in nursing homes (*n* = 31), 16% in care homes (*n* = 10), 13% in long-term care facilities (*n* = 8) and 20% in either mixed settings or settings described as residential aged care facilities or similar (*n* = 12). Sample size ranged from 1 to 100 long-term care facilities and duration of the intervention ranged from 4 weeks to 5 years. It was unclear in the majority of studies whether the intervention time period reported referred to the length of the study or the length of intervention delivery. A number of interventions were identified as shown in Table 3, which were categorized as either care based, care planning based, organizational multicomponent interventions, education/training or other.

**Table 3.** Interventions used in studies included in the review.

Category	Intervention	*n* = 65
Care based	Namaste Care Programme	2
	Comfort Care Rounds Strategy	1
	Compassion Intervention	1
	Joint working, that is, case conferencing, team working, integrated working between health care professionals and care home staff	5
	Other care based	2
Care planning based	Advance care planning (ACP) based	6
	ACP – Respecting Patient Choices	3
	ACP – ‘Let Me Decide’	2
	ACP – ‘We Decide’	1
Organizational multicomponent interventions	Gold Standards Framework for Care Homes	8
	Steps to Success programme	3
	Liverpool Care Pathway	3
	Care pathway or toolkit	4
	Other predefined, multicomponent intervention	3
Education and training	Staff education or training on improving palliative care	19
Other	Reduction in transfers, staff grief	2

Data extracted on the implementation of interventions of the studies included in the review are detailed in the Supplementary Material. In terms of facilitation, 85% interventions included some kind of facilitation (*n* = 55), 82% were externally facilitated (*n* = 53), 48% were internally facilitated (*n* = 29) and 40% were both internally and externally facilitated (*n* = 26). In 15% of interventions (*n* = 10), no form of facilitation was reported. In 97% of interventions, some kind of education component was involved; 8% (*n* = 5) delivered training online and 8% (*n* = 5) specifically involved providing training to health care professionals outside the long-term care facilities, such as physicians or paramedic emergency staff.

In terms of internal engagement, 97% of interventions reported staff members engaged within the long-term care facilities. In total, 23% (*n* = 15) of studies distinguished between registered nurses and care assistants, non-clinical staff or unregulated staff members, and 23% (*n* = 15) explicitly involved long-term care facilities managers. Residents and relatives were involved in 11% (*n* = 7) of studies. In terms of external engagement, 52% interventions reported some form of engagement (*n* = 34), usually with physicians or general practitioners; however, it would be unclear whether such joint working was already in place before the intervention.

Data were extracted on facilitators and barriers to implementation, including solutions to perceived barriers, for example, strategies to mediate staff turnover. The data were coded and categorized into nine sub-themes (presented below) which were identified acting as facilitators and/or barriers to implementing palliative care interventions in long-term care facilities. These could be categorized as falling into one of the three stages of the implementation processes: (1) establishing conditions to introduce the intervention, (2) embedding the intervention within day-to-day practice and (3) sustaining ongoing change. Quotes from the papers are used to illustrate the findings as shown in Table 4.

**Table 4.** Stages, themes and supporting quotes identified in the review.

Theme	Sub-theme	Example
Stage 1 – establishing conditions to introduce the intervention	Recognizing palliative care within the LTCF	‘Only 6 of the 14 facilities had consistently working Palliative Care Teams throughout the study period. These teams, in contrast to teams in the other 8 treatment nursing homes, were characterized by clear and shared mission, a sense that the team influenced residents’ care, and a perception of continued team sustainability. They also appeared to have a more tangible support from and involvement of their facility leaders including directors of nursing and administrators’ (p. 3).^41^
	Support from LTCF management	‘At site 1, improvements were made in pain assessment but not other measures. There were 3 different administrators during the 1 year pilot program. Despite initial interest, none of these administrators actively promoted palliative care and consequently, efforts to motivate staff to improve outcomes were hindered’ (p. 38).^42^
	Raising awareness among stakeholders	‘In our own project we found that involving residents and relatives in the decisions about implementation helped address staff concerns about the possible reluctance of the resident or their family member to participate in an ACP discussion. It provided an opportunity to emphasize that ACP discussions would become a routine practice with every resident so no individual resident would feel singled out’ (p. 148).^43^
Stage 2 – embedding the intervention within day-to-day practice	Locating the intervention within the current context	‘Overall, the time available for the NCP activities was less than anticipated. Two sessions a day was soon found to be too much for the staff to engage with, and the programme was reduced to one session held after lunch. While each session was to last for two hours, the complexity of getting all involved ready to start took longer than expected and this curtailed the duration of the activities in each session. Furthermore, although it is recommended that the NCP be held daily, in this care home it was only feasible to hold it Monday to Friday’ (p. 372).^44^
	Adopting a ‘whole home’ approach	‘Several nursing home managers have asked that we also train their non-clinical staff, who often become emotionally involved with residents, especially when these have been living in the home for a long time’ (p. 233).^45^
	Flexibility in implementation	‘The lack of continuity of staff was one of the most important factors affecting link nurse development. Staff shortages, high staff turnover and structuring the education around shift work were predominant features. Consequently, the delivery of education to suit different shifts had to be included. Attendance at educational sessions was therefore unpredictable’ (p. 239).^46^
Stage 3 – sustaining ongoing change	Ongoing opportunities for practice and reflection	‘Not all learners were equally ready to receive training at a particular level. For example, some less experienced care staff found it difficult to watch emotionally challenging content about death and dying on DVDs on their own. They preferred group work and discussions that could offer immediate debriefing. As stated by a trainer, the ability to be present during learning helped to address emotional reactions to the training’ (p. 275).^47^
	Appropriate selection of facilitators	‘Many facilitators reported that it was extremely important to provide a very clear outline of the commitment required from care homes in order to complete the programme. This was in terms of time allocated by managers for staff to complete the additional work needed and a requirement of attendance at the face-to-face sessions’ (p. 5).^48^
	Moving from intervention to routine practice	‘End of life care pathways are feasible mechanisms for delivering end of life care consistent with best practice. Strategies to facilitate acceptability by residential aged carew facility staff and GPs include incorporating end of life care pathways into existing standards and practices, and promoting awareness, education and accessibility’ (p. 109).^49^

LTCF: long-term care facilities.

## Stage 1 – establishing conditions to introduce the intervention

### Sub-theme 1 – recognizing palliative care within the long-term care facilities (*n* = 32)

The recognition of providing good-quality palliative care to residents as a priority by long-term care facilities managers and staff was a precursor to engagement with an intervention. In addition, an internal assessment acknowledging that palliative care within the setting could be improved was important in both supporting the initial intervention and sustaining change after implementation. An audit of the current practices at end of life within the long-term care facilities could be beneficial, as it allows staff to reflect on current practices and highlight areas where there is a need for improvement. Consequently, it provides a method of measuring future progress post-intervention by defining what good palliative care is within the context of the facility. Depending on facility organization and ownership, some facilities may have little or no communication with other long-term care facilities within the geographical area. Building long-term care facilities networks can provide shared support and learning opportunities, which may be of benefit to staff and managers alike, and allow benchmarking between facilities.

### Sub-theme 2 – support from long-term care facilities management (*n* = 45)

The review suggests that overt management support and enthusiasm to improve palliative care was extremely important, either through developing a vision for palliative care in the long-term care facilities or through supporting staff involvement by providing protected time and resource allocation for education and training. In particular, support for staff education sessions was paramount to ensuring high attendance. The establishment of a shared vision between management and facility staff regarding what the intervention is aiming to achieve facilitated implementation. Primarily, this could be achieved through either payment for attendance at sessions run outside the working day or allowing staff to attend sessions during their shift. Awarding certificates or continuing professional development credits were also used as incentives for staff to participate. Ensuring that facilities have the physical resources to complete the intervention are also important; this could range from having a space to conduct training in or access to the Internet for staff if training is being delivered online. In addition to the continuity of long-term care facilities staff, consistent long-term care facilities management able to promote the intervention was integral to success.

### Sub-theme 3 – raising awareness among stakeholders (*n* = 12)

Raising awareness of the aims and scope of the intervention to wider stakeholders outside of those delivering the intervention was highlighted in the review as it allowed for wider investment in improving palliative care, in terms of time and resources. Establishing the importance of palliative care among higher levels of management, such as commissioners and long-term care facilities administrators, facilitated implementation. The extent to which health care professionals from external settings were involved in the intervention varied depending on the context. At a minimum, an awareness of the intervention and its goals among health care professionals providing care to residents living in the long-term care facilities ensures that the intervention being delivered is congruent with the wider care of the resident.

In addition, raising awareness of the intervention with the residents and their families meant that changes in the delivery of palliative care were expected and the reasons for changes understood. An understanding of the intervention and its aims meant that residents and families did not feel that changes within the long-term care facilities were specific to the needs of an individual resident, but reflective of a facility-wide effort to improve care. In addition, family involvement increased awareness of palliative and end-of-life care within the long-term care facilities and facilitated discussions on treatment preferences.

## Stage 2 – embedding the intervention within day-to-day practice

### Sub-theme 4 – locating the intervention within the current context (*n* = 40)

The review suggests that a central characteristic of successfully implementing an intervention was the incorporation of the changes in the delivery of palliative care into the current practices within the facility. Without such an approach, there was a risk that the intervention would be unnecessarily adding to staff workload by duplicating processes or procedures that were already in place. Incorporation into existing practices and systems could range from adjusting documentation and record-keeping to developing how staff worked within the wider health care system. In cases where the intervention required involvement from wider health care professionals outside of the facility, adaptions were needed to develop existing relationships and current practices. Locating the intervention, therefore, requires first, an understanding of the current involvement of external professionals as part of understanding the context of the facility, and second, adaptions to the intervention to incorporate existing patterns of working.

### Sub-theme 5 – adopting a ‘whole home’ approach (*n* = 39)

A ‘whole home’ approach to change relates to developing an awareness of the intervention throughout the facility. Although the intervention may be specifically for staff undertaking certain roles, such as registered nurses or those providing clinical care, raising awareness of the intervention can improve an understanding of palliative care among all staff within the long-term care facilities. An all-encompassing approach is especially important in palliative care where residents or their family members may have conversations with staff who are not providing direct care, such as domestic or ancillary staff. In addition, clarifying how an intervention can be implemented by staff within their roles and responsibilities can build confidence, especially in non-clinical staff. Identifying staff members who have influence over others, or who are ‘informal leaders’ within the long-term care facilities and whose involvement in the study may inspire other staff members, can support this.

### Sub-theme 6 – flexibility in implementation (*n* = 37)

The review suggests that implementation of palliative care interventions can be hindered by a high turnover of staff in the facility. More than one staff member is needed for an intervention to be adopted into common practice; if there is a lack of continuity in staffing, this can be difficult to achieve. A ‘critical mass’ of staff who have completed the appropriate training and are motivated and supported in implementing changes is needed. Uneven participation, staff absences and high staff turnover are major barriers to achieving this, so maximizing opportunities to cascade knowledge and changes to practice between all staff is needed. Ensuring the intervention can be delivered flexibly, depending on the individual needs of the long-term care facilities, can improve implementation. This could be through the timing and frequency of education or training sessions, the mode of delivery (face-to-face or online) and the location of training, that is, internal or external. Aids to training, such as workbooks or decision aids, may also improve the integration of the intervention into every day practice, such as a resource folder or reference material that can be referred to as required and may serve as a training aid for new staff.

## Stage 3 – sustaining ongoing change

### Sub-theme 7 – ongoing opportunities for practice and reflection (*n* = 30)

The review highlighted the importance of developing opportunities for reflective debriefing time, staff discussion and confidence building through face-to-face workshops, role-play and on-the-job training. Although this strategy may support implementation of generic improvement initiatives, providing time and space for staff to talk openly about their feelings specifically towards delivering palliative and end-of-life care was highlighted as important. In some long-term care facilities, staff may not receive the emotional support they need, which can further hinder the improvement of palliative care. Reflection on practice could be achieved using examples that staff members can relate to, either through talking about experiences or through discussing the palliative care needs of a current resident. Workshops that are delivered face-to-face, role-play and on-the-job training can facilitate the transition from training to practice. In addition to reflecting on current palliative care practices, an assessment of the current levels of team working within the facility is required. In cases where team working within the facility is poor, interventions may be required, including training and guidance on wider elements of team working, development of communication skills and other ‘soft skills’ which may not be in place.

### Sub-theme 8 – appropriate selection of facilitators (*n* = 29)

The review highlighted the need for interventions to be either internally and/or externally facilitated. The review further suggested that facilitators (or trainers) should be identified appropriate to the number of residents in the facility, based on role and on their palliative care expertise. Whether or not facilitation is externally or internally provided, how facilitators will work with the facility to support the intervention and be trained and supported should be established early as part of the intervention. Internal facilitation requires appropriate selection of an existing staff member who can champion the intervention within the long-term care facilities. While an internal facilitator may have an understanding of the long-term care facilities, in terms of barriers to implementation and how they can be overcome, it may be difficult to manage the dual role and responsibility of being a staff member and internal facilitator. An external facilitator may have more clarity regarding their role and may have the ability to coordinate links with wider palliative care services; implementation may become reliant on the external facilitators visiting the long-term care facilities and may not be sustained once this is withdrawn. In larger long-term care facilities, a greater number of facilitators, either internal or external, will be needed to ensure that staff have access to the support they require to develop palliative care. In addition, facilitators should be identified as champions of palliative care possessing the ability to signpost less experienced staff members and aiding further education and development.

### Sub-theme 9 – moving from intervention to routine practice (*n* = 12)

The review clearly identified that successfully implementing an intervention requires its incorporation into existing practices in the long-term care facilities. Without such an approach, there was a risk that the intervention would be unnecessarily adding to staff workload by duplicating processes or procedures that were already in place. As part of the training or education element, communicating to staff members on how the new knowledge is going to be applied to routine care is important in changing practice. In some cases, this may require changing organizational structures or adapting the intervention to sit within current structures, for example, changing documentation to reflect new approaches. Consolidating and sustaining the changes made in the intervention post-delivery are seldom acknowledged in implementation studies. Data are limited regarding strategies to ensure sustainability; this is due to limited follow-up of long-term care facilities post-intervention, opportunities to retrain staff on an ongoing basis or as part of an induction and availability of funding to continue development roles or ongoing partnerships. These are beneficial when initiated as part of the original intervention.

## Discussion

### Main findings

This review aimed to identify the implementation strategies used in organizational level interventions to improve palliative care in long-term care facilities. It explored four implementation strategies: facilitation, education/training, and internal and external engagement. Based on the data reported in the papers that were included, nine themes were identified as potential facilitators and/or barriers to successful implementation of these interventions, which were then grouped into three development stages: establishing conditions to introduce the intervention, embedding the intervention within day-to-day practice and sustaining ongoing change.

The findings of the review have highlighted that the feasibility of implementing palliative care interventions is largely dependent on the context, and the extent to which delivery can be tailored to the individual needs of the facility, its staff and its residents. In addition, successfully implemented interventions were able to either improve or adapt to relatively poor existing conditions. These included poor communication between health professionals, long-term care facilities staff and families, high staff turnover and unsupportive management or a lack of leadership.

Palliative care interventions are increasingly complex, and exploring the implementation strategies that lead to changes in palliative care practice is a priority to inform future intervention development. This review categorized four implementation strategies: facilitation, education/training and internal and external engagement; however, the extent to which each strategy supports successful implementation is unclear. In previous systematic reviews on interventions that attempted to change staff practice to improve long-term care facilities resident outcomes and on implementing advance care planning in nursing homes, similar barriers and facilitators to implementing interventions were identified as those found in this review.^23,24^

The Promoting Action on Research Implementation (PARIHS) framework has been used to guide implementation of interventions in long-term care facilities and focuses on the interplay between the evidence being introduced, contextual characteristics of the setting and facilitation.^25^ In the Facilitating Implementation of Research Evidence (FIRE) study, a cross-country comparison of two facilitation approaches in 24 long-term nursing care units, an improvement based and practitioner inquiry approach, against standard dissemination of clinical guideline recommendations found no significant differences between the two approaches.^26^ Similar barriers were identified as those discussed in this paper, such as issues with recruitment and retention of internal facilitators, issues in preparing facilitators for the role and application of facilitation knowledge, skills and tools.^27^ The evaluation of a standardized education intervention of Mekki et al.^28^ to reduce restraint and agitation in residents living with dementia in nursing home residents, using the PARIHS framework, identified that while success required interplay between the three elements of the framework, a specific focus on leadership was needed for successful intervention.

In addition to the extent to which different implementation strategies contribute to success, how these strategies can be utilized requires further examination. In a systematic literature review on the role, use and preparation of champions within nursing homes to inform quality improvement approaches, Woo et al.^29^ found that although all the included studies suggested that implementing nurse or aid champions in their quality improvement initiatives were important facilitators of success, how the champions were selected and trained in their role was underreported. Kinley et al.^30^ found that nursing homes that received high facilitation and action learning to implement the Gold Standards Framework for Care Homes were more likely to be accredited than those with high facilitation only. How we measure implementation, in terms of fidelity and sustainability, in addition to the intended outcomes of the intervention, also requires further thought.

Research on palliative care interventions in long-term care facilities has a dual purpose; first it determines whether an intervention is effective in improving care, and second, it explores whether an intervention can be used in a real-world setting. An integrative review of effective implementation strategies previously used to improve the organization of palliative care in adults across care settings identified a number of approaches: feedback, educational strategies, process mapping, feedback, multidisciplinary meetings and multifaceted interventions.^31^ While there is potential for learning from other settings within the health system, exploring what works specifically in palliative care in long-term care facilities is crucial to move from evidence to changing practice.

### Strengths and limitations

This scoping review has followed the methodological steps described in the Arksey and O’Malley^16^ framework and incorporated enhancements of the method discussed by Levac et al.,^32^ including ensuring adequate clarity on the scope of the review, using the research question to guide decision-making and adopting an iterative approach to study selection and data charting.^33^

A strength of this review is the inclusion of all study designs, which has allowed data to be extracted on intervention studies using both quantitative and qualitative approaches. The inclusion of studies using qualitative methods greatly added to the understanding of facilitators and barriers to implementation, as themes emerging through data collection were reported as results in addition to insights from the study authors.

The review was restricted to studies published in English between 2007 and 2018, meaning studies outside these limits were missed. However, for the purpose of the review, the aim was to produce an overview of the research area focusing on breadth rather than depth of understanding, which has been achieved. In addition, guidance from methodological papers on scoping review reporting standards^16,19,22^ has provided a framework to add methodological integrity to the review, despite not being a systematic review. Implementation may be reported in grey literature; however, this is harder to access, often published as reports within national bodies.

A limitation of this review is that data on reporting of fidelity of implementation within studies, if reported, were not extracted, and characteristics of implementation were not linked to reported outcomes. In addition, the implementation strategies identified in this scoping review have predominantly been used in the context of funded research. However, outcome measures of staff perception, competence and confidence may not lead to actual changes in practice. Data on the long-term sustainability of an intervention can be difficult to capture within limited funding time periods for research studies. This can make it difficult to explore how implementation strategies can support intervention longevity. In addition, it is unclear how variation between wider health care systems within which these interventions were implemented may affect successful implementation.

The final stage of the Arksey and O’Malley^16^ framework, consultation with stakeholders to provide insights into the findings, was also not completed. The inclusion of this stage could have allowed an opportunity to provide further understanding of the findings from long-term care facilities staff and managers.

### Implications for further research

Long-term care facilities are complex and challenging environments in which to enact change, and developing an understanding of approaches which facilitate implementation requires attention. As discussed, further research is needed to identify the contribution of individual implementation strategies as well as the interplay between them. In the development of palliative care interventions, adopting a theory of change tailored to the aims of the intervention that can guide implementation may be beneficial in delivering the intervention within a real-world scenario. In future, better reporting of implementation strategies and their successes is needed to further inform the development of palliative care interventions. Checklists, such as the template for intervention description and replication (TIDIER) checklist, could be adopted as reporting guidelines for intervention studies.^34^ In addition to reporting implementation, implementation fidelity in palliative care is also underreported; strategies to improve implementation fidelity have been proposed.^35^

This review specifically focused on long-term care facilities taking part in research studies or evaluations, all of which had an initial willingness from within the facility to actively receive a palliative care intervention and had some form of involvement from a research team to collect data, at a minimum. It is unclear how implementation may differ without the involvement of a research team or without an evaluation or audit process. Separating barriers and facilitators to implementation with that of the research process, such as recruitment, retention and attrition, is an additional complexity, as is how to engage long-term care facilities who are unwilling to take part in such studies.^36^

Information on implementation is seldom reported in detail, creating difficulties in establishing the elements of an intervention that is being newly delivered or being incorporated in current practice. For example, while some studies have reported multidisciplinary team meetings as part of their intervention, it is unclear whether such meetings were in place before the intervention and to what extent. Further research could also explore the cost-effectiveness of interventions and their sustainability after external facilitation has ended.

### Implications for practice

The potential for interventions to improve palliative care in long-term care facilities is well-documented in previous research; however, implementation of such interventions is under reported. This review has highlighted the difficulty of separating characteristics of palliative care interventions with their implementation. In some studies, the challenges encountered in implementation may be inherent to the nature of the intervention, for example, staff members fearing engaging in advance care planning discussions with residents.^37^ A key finding of the review is the need for palliative care interventions to support wider skills, such as supporting team work, in addition to improving palliative care knowledge. Facilitators include strong leaderships within the facility; availability of external facilitation and a culture of learning indicate that future research should explore support for managers to develop a culture of palliative care learning and reflection among staff members. These facilitators move interventions from understanding to sustained changes in practice and improvement in palliative care. Drawing on wider implementation literature, specifically theories of implementation from other areas of health care, could inform implementation within this area.^38–40^

### Implications for policy

The review has identified a number of factors associated with implementing palliative care initiatives in long-term care facilities at an organizational level; the majority of which are understandably located at an organizational level. It is unclear from these findings how the promotion of palliative care at regional, national and international level can support change at an organizational level. Fully integrated palliative care within long-term care facilities will require the establishment of minimum palliative care competencies for long-term care facilities staff and appropriate regularity frameworks and guidance, which will require a multilevel approach.

## Supplemental Material

Implementing_palliative_care_in_LTCFs_-_Supplementary_File_30092019 – Supplemental material for Strategies for the implementation of palliative care education and organizational interventions in long-term care facilities: A scoping review Click here for additional data file. Supplemental material, Implementing_palliative_care_in_LTCFs_-_Supplementary_File_30092019 for Strategies for the implementation of palliative care education and organizational interventions in long-term care facilities: A scoping review by Danni Collingridge Moore, Sheila Payne, Lieve Van den Block, Julie Ling and Katherine Froggatt in Palliative Medicine
