# Supplementary material for: Strategies for the implementation of palliative care education and organizational interventions in long-term care facilities: A scoping review
Source: Palliat Med. 2020 Feb 3;34(5):558–70. doi: 10.1177/0269216319893635 (PMC7222696; doi:10.1177/0269216319893635)
Supplement: Implementing_palliative_care_in_LTCFs_-_Supplementary_File_30092019 – Supplemental material for Strategies for the implementation of palliative care education and organizational interventions in long-term care facilities: A scoping review [file Implementing_palliative_care_in_LTCFs_-_Supplementary_File_30092019.docx]

1. exp Nursing Homes/
2. exp Homes for the Aged/
3. "care home*".ti,ab.
4. "nursing home*".ti,ab.
5. "nursing care home*".ti,ab.
6. "nursing facilit*".ti,ab.
7. "residential home*".ti,ab.
8. "residential care".ti,ab.
9. "residential long term care".ti,ab.
10. "institutional* care*".ti,ab.
11. ("long term" adj1 "care facilit*").ti,ab.
12. ("long term" adj1 "care residen*").ti,ab.
13. ("long term" adj1 "care institution*").ti,ab.
14. ("long term" adj1 "institution* care*").ti,ab.
15. ("institution*" adj1 "long term care*").ti,ab.
16. 1 or 2 or 3 or 4 or 5 or 6 or 7 or 8 or 9 or 10 or 11 or 12 or 13 or 14 or 15
17. exp Palliative Care/
18. exp [Palliative Medicine](http://ovidsp.tx.ovid.com.ezproxy.lancs.ac.uk/sp-3.27.1a/ovidweb.cgi?&Controlled+Vocabulary=Mapping%7c0&Return=mapping&S=EFFAFPKHLIDDJNJJNCFKPAOBHAHAAA00)/
19. "palliative care".ti,ab.
20. "palliative support".ti,ab.
21. "palliative medicine".ti,ab.
22. "end of life".ti,ab.
23. "supportive care".ti,ab.
24. exp Terminal Care/
25. "terminal".ti,ab.
26. exp Hospice Care/
27. "hospice care".ti,ab.
28. exp Resuscitation Orders/
29. exp Advance Care Planning/
30. "advance care planning".ti,ab.
31. "ACP".ti,ab.
32. exp Advance Directives/
33. "advance directive*".ti,ab.
34. “Gold standards framework".ti,ab.
35. "GSF".ti,ab.
36. "Steps to success".ti,ab.
37. "Route* to success".ti,ab.
38. 17 or 18 or 19 or 20 or 21 or 22 or 23 or 24 or 25 or 26 or 27 or 28 or 29 or 30 or 31 or 32 or 33 or 34 or 35 or 36 or 37
39. "Implement*".ti,ab.
40. "Effect*".ti,ab.
41. "Coordinat*".ti,ab.
42. "Facilit*".ti,ab.
43. "Strateg*".ti,ab.
44. "Improv*".ti,ab.
45. "Review*".ti,ab.
46. "Manag*".ti,ab.
47. "Involv*".ti,ab.
48. "Integrat*".ti,ab.
49. "Multi?disciplinary ".ti,ab.
50. "Multi?professional".ti,ab.
51. "Debreif*".ti,ab.
52. "Train*".ti,ab.
53. "Educati*".ti,ab.
54. "Support*".ti,ab.
55. "Deliver*".ti,ab.
56. 39 or 40 or 41 or 42 or 43 or 44 or 45 or 46 or 47 or 48 or 49 or 50 or 51 or 52 or 53 or 54 or 55
57. 16 and 38 and 56
58. limit 57 to yr="2007 -Current"
59. Aged/
60. 58 and 59

**Supplementary material 1: Example search strategy – Ovid Medline**

| **Supplementary material 2: Overview of interventions identified** | | | | | |
| --- | --- | --- | --- | --- | --- |
| **Author / year**  **Country**  **Study design** | **Aim of study** | **Setting**  **Sample size**  **Participants** | **Study / intervention duration** | **Description of intervention** | **Outcome measures and findings** |
| Aasmul et al, 2018 (1)    Norway  Cluster randomized controlled trial | To describe the content of advance care planning (ACP) in the COSMOS study and the evaluation of the implementation process of the intervention in Norwegian nursing homes. | Nursing homes n=37  765 patients | 4 months/  NR | Education programme to learn early and repeated communication with patients and families and to implement ACP. | **Outcome measures:** Feedback during midway seminars and individual patient logs.  **Findings:** The patient logs showed that ACP was successfully implemented in 62% (n=183) of patients. The staff emphasized the clear communication of the relevance of ACP addressed to leaders and staff as important facilitators, along with the clearly defined routines, roles and responsibilities. Identified barriers included lack of competence, perceived lack of time, and conflicting culture and staff opinions. |
| Agar et al 2017,  Luckett et al, 2017 (2, 3) *  Australia  Cluster randomized controlled trial | To explore the benefits of facilitated case conferencing and the Palliative Care Planning Coordinator (PCPC) role, as well as factors influencing implementation, as perceived by PCPCs themselves and the health professionals who participated. | Nursing homes n=20/n=10  131 residents/40 staff members | 18 months/ 18 months | Facilitated case conferencing organised by PCPCs. | **Outcome measures:** Family rated quality of end of life care (End of life Dementia [EOLD] Scales), nurse-rated EOLD scales, resident quality of life (Quality of Life in Latest age Dementia) and quality of care over the last month of life (pharmacological/ non-pharmacological palliative strategies, hospitalization or inappropriate interventions). Semi structured interviews focused on perceptions regarding the impacts of facilitated case conferencing and barriers and facilitators to this as a means of improving resident care.  **Findings:** Facilitated case conferencing facilitates a palliative approach to care. Perceived benefits of facilitated case conferencing included better communication between staff and families, greater multi-disciplinary involvement in case conferences and care planning, and improved staff attitudes and capabilities for dementia palliative care. Key factors influencing implementation included staffing levels and time; support from management, staff and physicians; and positive family feedback. |
| Amador et al, 2016 (4)  United Kingdom  Mixed methods study | To report on the qualitative component of a mixed method study aimed at evaluating an organisational intervention shaped by Appreciative Inquiry to promote integrated working between visiting health care practitioners and care home staff. | Care homes n=3  4 staff members in each care home (care home manager, deputy manager, GP, district nurse) | 6 months/  18 months | Appreciative Inquiry (Social Identity Approach). | **Outcome measures:** Semi structured interviews.  **Findings:** The intervention supported integrated working through the development of a common group identity built on shared views and goals, but also recognition of knowledge and expertise specific to each service group, which served common goals in the delivery of end of life care. It supported the development of context specific practice innovations and the introduction of existing end of life care tools and frameworks. |
| Ampe et al, 2017 (5) *  Belgium  Quasi-experimental pre-test/post-test study with an intervention and control group | To evaluate the influence of the intervention ‘we DECide – Discussing End of life Choices’ on the policy and actual practice of ACP in nursing home dementia care units and to investigate barriers and facilitators for the implementation of we DECide. | Nursing homes n=18  90 nursing home staff members | 8 months/  4 weeks | ‘’We DECide’, an educational intervention for nursing home staff on shared decision‐making in the context of ACP for residents with dementia. | **Outcome measures:** Compliance with best practice of ACP policy, ACP practice and degree of involvement of residents and families in conversations, perceived barriers and facilitators for the implementation of shared decision making in ACP practice.  **Findings:** ACP was significantly more compliant with best practice after the intervention, however was not discussed more frequently, nor were residents and families involved to a higher degree in conversations after the intervention was implemented. Barriers to realizing ACP included staff’s limited responsibilities. |
| Andrews et al, 2009 (6)  Australia  Action research | To explore how staff members from residential dementia special care units could develop strategies to support a palliative approach to care following the Guidelines for Palliative approach in residential aged care. | Residential dementia special care unit n=1  5 staff members/  10 family members | 18 months/  18 months | Action research involved semi-structured interviews to staff, residents and family members, resulting in an information package. provided to family caregivers. | **Outcome measures:** Evaluation questionnaires.  **Findings:** Staff accessed evidence-based resources and developed strategies to address the information needs of family members. Evaluation by family members showed a positive response to the information provided. |
| Arcand et al, 2009 (7)  Canada  Pre-test/post-test study | To assess the impact, in terms of family satisfaction with end of life care, of a nursing home pilot educational program for nursing staff and physicians on comfort care and advanced dementia. | Nursing home n=1  NR staff members  21 relatives of residents who died of dementia (post intervention) | NR/  4 months | Educational program and a booklet for staff, and optionally to families. | **Outcome measures:** After death bereaved family interview/nursing home version. Educational program providing an information booklet.  **Findings:** Scores on satisfaction with pain control, emotional support, treating patient with respect, and information on what to expect while patient was dying improved post intervention. There were no statistical differences between the two groups, although the post-intervention group expressed greater satisfaction in communication with the health care team and greater global satisfaction with care. |
| Badger et al, 2007, Badger et al, 2009, Badger et al, 2012 (8-10)  United Kingdom  Pre-test/post-test study and qualitative case study | To evaluate the impact of a training programme to improve end of life care in nursing homes, on collaboration between nursing home staff and other health practitioners. | Nursing homes n=95  n=44 included in the final analysis  NR managers, staff, residents and family | 8 months/  8 months | Gold Standards Framework in Care Homes. | **Outcome measures:** After death analysis and case study methodology.  **Findings:** Post intervention, more homes had a register of residents’ end of life care needs and were using guidelines to help identify residents. Hospital deaths reduced from 18% to 11% and in crisis hospital admissions from 38% to 26%. Improved collaborations were anticipated by 31% of managers. Staff reported increased knowledge of end of life care, and enhanced confidence, which in turn resulted in improved communication and collaboration. Key improvements included better care planning, communication, staff confidence, collaboration with others and significantly reduced crisis hospital admissions and a reduction in hospital deaths. |
| Beck et al, 2012,  Beck et al, 2015 (11, 12)  Sweden  Quasi-experimental pre-test/post-test study and semi structured interviews | To compared the efficacy of facilitated case conferencing versus usual care in improving end of life care for persons with advanced dementia living in nursing homes.  To describe the nurse assistants’ experience of how an intervention with a palliative care approach had influenced them in their work in residential care for older people. | Residential care facilities n=9/n=3  82 nurse assistants,  9 managers/ registered nurses | 12 months/  7 months | Circle sessions interspersed with workshops, semi-structured individual interviews. | **Outcome measures:** Job satisfaction questionnaire, Psychosocial Aspects of Job Satisfaction scale, Strain in Dementia Care Scale, Stress of Conscience Questionnaire, Leadership Behaviour Questionnaire.  **Findings:** After the intervention, nurse assistants increased awareness of, and respect for, the needs of the residents and their relatives, increased understanding of their own importance in the encounter with residents and relatives. Increased openness and understanding among colleagues. Nurse assistants described lack of resources and supportive leadership. Job satisfaction of nurse assistants decreased and they perceived the leadership more negatively than before the intervention. |
| Blackford et al, 2007 (13)  Australia  Evaluation | To report on the lessons learnt from the implementation of the Respecting Patient Choices intervention and identify strategies which foster sustainability of ACP. | Residential aged care facilities n=17  1000 frail elderly  14 staff | 18 months/  NR | Respecting Patient Choices. | **Outcome measures:** Audit of the ACP documentation and medical records of those residents who had died, semi structured interviews to staff in facilities.  **Findings:** Post intervention, 51% residents had been introduced to Respecting Patient Choices, with an uptake of 52%. Governance structure, educational processes, resident documentation, and quality audit processes as well as communication across organisations ensured sustainability,. as did an audit of current practices, ACP process, maintaining ongoing ACP education and support for staff, documentation and medical records, promoting continuity in ACP-information transfer and quality processes. |
| Booth et al, 2014 (14)  United Kingdom  Evaluation of educational interventions | To explore advantages and disadvantages of three initiatives to delivering end of life education to care homes in southeast England. | Care homes n=11  14 managers or deputy managers | 24 months/  24 months | Action learning project. | **Outcome measures:** Hennessey and Hicks Training Needs Tool, confidence and competence questionnaires and focus groups.  **Findings:** Participants were empowered as managers and role models in end of life care. |
|  |  | Care homes n=18  38 staff members | 8 months/  8 months | Six Steps to Success programme. | **Outcome measures:** Audits of knowledge, skill and confidence, post-death information, and care home quality markers, accreditation.  **Findings:** Results were positive and encouraging. |
|  |  | Care homes n=23  70 students | 12 months/  12 months | Gold Standards Framework for Care Home. | **Outcome measures:** Accreditation.  **Findings:** All homes completed the first programme, but only one undertook accreditation. Others decided to ensure the programme was fully embedded prior to accreditation in 2014. |
| Brajtman et al, 2012 (15)  Canada  Pilot study/evaluation | To test an educational intervention about end of life delirium for inter-professional teams. | LTCF n=11  22 nursing, rehab, social workers | NR | Educational intervention. | **Outcome measures:** Inter-professional collaborative competencies attainment survey, and the We Learn seven-point Likert scale to measure overall satisfaction in an inter-professional education activity.  **Findings:** Participants at hospice and long-term facilities gave high satisfaction ratings to the overall content, structure, service and outcomes of the intervention. At long-term facilities, significant increases were found for all competencies; however, there was no significant change after intervention. |
| Brännström et al, 2005 (16)  Sweden  Evaluation | To compare the effects of the Liverpool Care Pathway (LCP) for the Dying Patient and usual care on patients’ symptom distress and well-being during the last days of life, in residential care homes. | Residential care homes n=19  135 family members of deceased participants | 15 months/  15 months | Liverpool Care Pathway. | **Outcome measures:** Edmonton Symptom Assessment (ESAS), Views of Informal Carers – Evaluation of Service (VOICES) questionnaire.  **Findings:** Shortness of breath and nausea were significantly reduced. A statistically significant improvement in shortness of breath was also found on the VOICES questionnaire. |
| Campion et al, 2016 (17)  United Kingdom  Implementation study | To describe an innovative model of education and training for nursing home staff ... to improve end of life care for residents. | Nursing homes n=33  NR Nursing home staff  NR nursing home residents | NR/NR | Education and training including clinical rounds, advice and guidance, communication, and care co-ordination. | **Outcome measures:** Place of death, contact with the ambulance service, transfers to hospital.  **Findings:** Post implementation, 85% died in their preferred place, 18% died in an acute hospital setting. Introduction of ‘Coordinate my Care’ records coincided with a reduction in the number of contacts with the ambulance service and of transfers to hospital - there were 8.3% fewer calls from nursing homes to the ambulance service. There was also a reduction in the number of ambulance transfers of nursing home residents to hospital. |
| Chapman et al, 2018 (18)  Australia  Quasi-experimental design | To introduce a model which provides proactive specialist palliative care to supplement the palliative approach to residents’ care. | Residential facilities n=4  104 residents | 9 months/  6 months | Palliative Care Needs Round, including monthly onsite clinical meeting. | **Outcome measures:** Number and length of hospitalisations, preferred place of death and location of death.  **Findings:** The intervention was associated with a reduction in the length of hospital stays and a lower incidence of death in the acute care setting. Rates of hospitalisation were unchanged on average, length of admission was reduced by an average of 3.22 days (p<0.01 and 95% CI −5.05 to −1.41), a 67% decrease in admitted days. |
| Chisholm et al, 2017, Hanson et al, 2016, Hanson et al, 2017 (19-21)  USA  Cluster randomized controlled trial  Evaluation | To understand nursing home staff perceptions of adoption and sustainability of the Goals of Care video decision aid for families of residents with advanced dementia.  To describe the Goals of Care cluster randomized trial and the methods used to monitor and promote fidelity to a goals of care decision aid intervention delivered in nursing homes.  To test a Goals of Care decision aid intervention to improve quality of communication and palliative care for nursing home residents with advanced dementia. | Nursing homes n=22/n=11  151 family decision-makers and residents dyads  94 nursing home staff (nurses, social works, therapists, nutritionists) | 18 months/  NR | Two-component intervention: a video decision aid about goals of care choices and a structured decision-making discussion with the nursing home care plan team. | **Outcome measures:** Quality of communication and decision making using the Quality and Communications questionnaire, toolkit ACP Problem score, treatment plan, family satisfaction with care, patient comfort, patient quality of life, hospice referral, and hospitalizations, family report of concordance with clinicians on the primary goal of care, family ratings of symptom management and care, palliative care domains in care plans, Medical Orders for Scope of Treatment (MOST) completion and hospital transfers.  **Findings:** Key supports for implementation included design features that aligned with nursing home practice, efficient staff training, and a structured guide for goals of care discussions between family decision-makers and staff. Family decision makers reported better quality of communication and better end of life communication with clinicians. Clinicians were more likely to address palliative care in treatment plans, use Medical Orders for Scope of Treatment, and less likely to send patients to the hospital. Family ratings of treatment consistent with preferences, symptom management, and quality of care did not differ. Residents in the intervention group had more palliative care content in treatment plans, MOST order sets, and half as many hospital transfers. Nursing home staff reported high ratings for adoption and sustainability of the Goals of Care intervention. On a scale from 1 to 6, staff perceived the Goals of Care intervention as relatively advantageous (mean 5.09), compatible with practice (mean 5.01) and easy to use (mean 5.16), indicating strong potential for adoption. |
| Cornally et al, 2015 (22)  Ireland  Focus groups | To evaluate the systematic implementation of the ‘Let Me Decide’ advance care directive and palliative care education programme. | Nursing homes n=3  15 clinical nurse managers / 2 directors of nursing | 2 years/  NR | ‘Let Me Decide’ - advance care-planning programme. | **Outcome measures:** Impact on quality of care, nurses’ knowledge, ACP uptake rates, compliance with resident’s wishes at end of life and barriers to implementing the programme.  **Findings:** The main benefits included enhanced communication and staff morale, changing the care culture, promoting preference-based care and avoiding crisis decision making. The main challenges reported by staff included establishing capacity among residents and indecision. |
| Cox et al, 2017 (23)  United Kingdom  Exploratory mixed methods design with pre and post intervention evaluation | To increase the confidence and competence of care home staff in end of life care; and enable more residents the opportunity to experience end of life care in their care home rather than an acute setting. | Residential care homes, nursing homes n=12 (4 RCH, 2 NH)  NR | 6 months/  3 months | End of Life Care toolkit. | **Outcome measures:** Staff confidence and competence, number of residents experiencing end of life care in an acute setting.  **Findings:** Following the intervention, there was a trend for staff to report feeling more supported both in terms of emotional and clinical support within the care home and feeling able to source external support. Staff confidence in managing pain management, addressing anxiety, nausea and vomiting and mouth care increased post intervention, however this trend did not reach statistical significance. A comparison of a 5-month period before and after the intervention indicated a 59% reduction in the number of residents who died in the local hospital from the six participating care homes in comparison to a 21% reduction from six comparison care homes who had not received the intervention. |
| Cronfalk et al, 2015 (24)  Sweden  Focus groups | To describe nursing home staff’s attitudes to three competence-building programs in palliative care. | Nursing homes  n=20  118 staff members (registered nurses, enrolled nurses, care assistants) | 1 year /  NR | Education of one to two persons per ward. | **Outcome measures:** Focus groups, experiences of competence based programmes and palliative care, tension between different professions, encounter older people's dying and death.  **Findings:** Attitudes toward the intervention were positive independent of their design or content. Enrolled nurses and care assistants felt that they carried out advanced care without the necessary theoretical and practical knowledge. Further, the results also suggest that lack of support from ward managers and insufficient collaboration and of a common language between different professions caused tension in situations involved in caring for dying people. |
|  |  | Nursing homes  n=11  363 staff members (registered nurses, enrolled nurses, care assistants) | 1 year /  NR | Separate seminars for different with a focus on the principles of palliative care. |  |
|  |  | Nursing homes  n=6  371 staff members | 1 year /  NR | Seminars introducing the LCP. |  |
| Farrington, 2014 (25)  United Kingdom  Pre-test/post-test study and semi structured interviews | To evaluate whether a blended e-learning training programme generate a positive change in participants’ understandings of, and confidence in delivering end of life care in care homes and identify the main barriers to translating new understandings into practice. | Nursing homes / residential care homes n=1  14 health care assistants/ 6 administrative staff | 8 months/  NR | The ‘ABC’ course, a blended e-learning programme (face-to-face facilitated workshops alongside online content). | **Outcome measures:** Staying Healthy Assessment questionnaire, a free text questionnaire, audit of clinical notes for deceased residents, semi-structured interviews.  **Findings:** Improvements in participants’ confidence in delivering end of life care. The questionnaire showed an increase in confidence in assessment and care planning, symptom management and well-being, communication, and ACP/ end of life tools. The overall average improvement in mean confidence levels was 0.8, representing a 28.7% advance in confidence across all competency areas. Several barriers were encountered, including uneven participation, the absence of mechanisms for disseminating new insights and knowledge within the home, and a widespread perception that nurses' professional dominance in the home made sustainable change difficult to enact. |
| Fernandes, 2008 (26)  Australia  Pre-test/post-test study | To examine the process of how residents’ end of life care wishes are recorded and to ensure the implementation of an advance care plan is performed according to the best available evidence. | Long term care facility (LTCF) / residential aged care places (RACP) n=100 LTCF /  n=1 (RACP) | 5 months/  NR | The Getting Research into Practice process of the Practical Application of Clinical Evidence System program. | **Outcome measures:** Audit of current practice.  **Findings:** Compliance with five evidence-based audit criteria on advance care planning, pre- and post-implementation of best practice increased from 77% to 100%.  The barriers identified for ACP included deficits related to the knowledge and education of residents, families and staff members, and issues related to administration and documentation, and concerns that any implementation process would not be sustainable. |
| Finucane et al, 2013 (27)  United Kingdom  Evaluation | To sustain the results achieved following the initial Gold Standards Framework in Care Homes project using a lower level of care home support. | Nursing homes n=7  132 residents  16 key champions (care assistants and trained staff nurses-original project)  3 key champions (following project) | 20 months/  NR | Gold Standards Framework in Care Homes. | **Outcome measures:** Do Not Attempt Cardiopulmonary Resuscitation documentation in place, proportion of deceased residents where Do Not Attempt Cardiopulmonary Resuscitation documentation completed, proportion of residents with any form of anticipatory care plan in place, proportion of residents known to have died on the adapted LCP, inappropriate hospital deaths and hospital deaths.  **Findings:** Increases in the proportion of deceased residents with an anticipatory care plan in place, the proportion of those with Do Not Attempt Cardiopulmonary Resuscitation documentation in place and the proportion of those who were on the LCP when they died. Furthermore, there was a reduction in inappropriate hospital deaths of frail and elderly residents with dementia. |
| Frey et al, 2017 (28)  New Zealand  Pre-test/post-test study and interviews | To explore the impact of Supportive Hospice Aged Residential Exchange for staff. | Residential care facilities n=2  58 registered nurses and health care assistants (questionnaire)  11 registered nurses and healthcare assistants, managers, hospice nurses (interviews) | 6 months/  NR | Supportive Hospice Aged Residential Exchange. | **Outcome measures:** Staff survey, including the Brief Screen depression measure and the Empowerment Scale, manager and staff interviews.  **Findings**: Results indicate that the intervention overall is seen as a success, especially in relation to advanced care planning documentation. Relationships between hospice and facility staff, and consequently facility staff and residents are seen as the key to the success of the project. Staff survey results indicated increased confidence in palliative care delivery and decreased depression. Key lessons learnt from for the development of any palliative care intervention within aged residential care include the importance of reciprocal learning, as well as the necessity of a strong partnership with key stakeholders. |
| Garden et al, 2016 (29)  United Kingdom  Evaluation | To provide guidance for others wishing to set up a similar service. The two objectives were to examine steps required to put this programme into practice, and to demonstrate the effects of doing so. | Care homes n=7  107 residents with dementia | 24 months/  24 months | Bromhead Care Home Service - Education programme based on the Stop Delirium! Material. | **Outcome measures:** Staff confidence, carer satisfaction, place of death, hospital admission.  **Findings**: Marked improvements in staff confidence were seen in recognition (64%), prevention (67%) and management (60%) of delirium which were all highly significant (p<0.01). There were also marked improvements in confidence levels in recognition (55%, p=0.0005) and management (48.4%, p=0.0039) of dysphagia with more modest improvement in knowledge of signs of dysphagia in dementia. High levels of carer satisfaction; 92% carers rated the service >9/10. Admissions fell by 37% from baseline in the first year and 55% in the second and third years. All but one resident died in the preferred place of care. |
| Giuffrida, 2015 (30)  USA  Evaluation | To describe the development of two innovative programs whose goals were to increase the number of residents receiving palliative care, increase the number of completed advance directives, reduce re-hospitalizations, and increase hospital referrals to the nursing home for palliative care. | Nursing and rehabilitation facility n=1  NR | NR/NR | Comprehensive palliative care program. | **Outcome measures:** Residents on palliative care, number of rehospitalisation’s, residents with health care proxies, residents with Do Not Resuscitate orders, residents with feeding tubes.  **Findings**: Post intervention, the number of residents on palliative care increased from 5% to 25%, re-hospitalization rates decreased from 17.4% to 15.2%, residents with health care proxies increased from 65% to 69%, residents with DNR orders increased from 64% to 73%, residents with feeding tubes declined from 24% to 14%. |
| Hall et al, 2011 (31)  United Kingdom  Evaluation - qualitative methods | To explore the views of care home staff, residents and their families on the benefits of and barriers to implementation of the Gold Standards Framework for Care Homes, to inform the development of palliative care interventions in care homes for older people. | Care homes n=9  26 staff members (9 care home managers  8 nurses  9 care assistants)  11 residents  7 family members | From 3 to 26 months/from 3 to 26 months | Gold Standards Framework for Care Homes. | **Outcome measures:** Semi-structured interviews.  **Findings:** Perceived benefits included improved symptom control and team communication; finding helpful external support and expertise; increasing staff confidence; fostering residents’ choice and boosting the reputation of the home. Perceived barriers included increased paperwork; lack of knowledge and understanding of end of life care; costs; and gaining the cooperation of GPs.  Although staff described the benefits of supportive care registers, coding predicted stage of illness and ACP, which included improved communication, some felt the need for more experience of using these, and there were concerns about discussing death. |
| Hasson et al, 2008 (32)  United Kingdom  Evaluation - qualitative study | To explore link nurses’ views and experiences regarding the development, barriers and facilitators to the implementation of the role of palliative care in the nursing home. | Nursing homes n=10  14 link nurses | 3 years/NR | Palliative care educational programme and link nurse role. | **Outcome measures:** Focus groups.  **Findings:** The link nurse system shows potential to enhance palliative care within nursing homes. However, link nurses experienced a number of difficulties in implementing education programmes. Facilitators of the role included external support, monthly meetings, access to a resource file and peer support among link nurses themselves. Lack of management support, a transient workforce and lack of adequate preparation for link nurses were barriers to fulfilling this role. |
| Hewison et al, 2008 (33)  United Kingdom  Case study approach /  evaluation - qualitative study | To report on how teamwork is perceived and managed in homes after the introduction of the Gold Standards Framework for end of life care in care homes, with particular emphasis on the relationship between teamwork and organisational and practice change. It explores two key areas: perceptions of staffing levels and team working in nursing homes. | Nursing homes n=95  n=9 (interviews)  14 managers (telephone interviews)  61 staff (group interviews)  7 residents, 3 relatives (face-to-face interviews) | NR/NR | Gold Standards Framework for Care Homes. | **Outcome measures:** Team working questionnaire, interviews face-to-face, group and telephone, audit data and direct observation of the Gold Standards Framework in action.  **Findings:** Teamwork is central to the successful introduction of the Gold Standards Framework in Care Homes. Good staffing levels and management support were key factors in homes where the Framework became established. Organisations wishing to implement such programmes should assess the quality of teamwork and may need to address this first. |
| Hickman et al, 2016 (34)  USA  Implementation study | To describe processes and preliminary outcomes from the implementation of a systematic ACP intervention in the nursing home setting. | Nursing homes n=19  2,709 residents  25 nurses (registered nurses, nurse practitioners) | 17 months/ NR | Advanced Care Planning, using a structured interview guide. | **Outcome measures:** Advanced care planning conversations  **Findings:** The intervention resulted in a change in documented treatment preferences for 69% (504/731). The most common change (87%) was the generation of a Physician Orders for Scope of Treatment form. The most frequently reported barrier to ACP was lack of time. |
| Ho et al, 2016a,  Ho et al, 2016b (35, 36)  China  Evaluation | To describe systematically the development and implementation mechanisms of a novel Dignity-Conserving End of life Care model.  To critically examine the underpinnings of palliative long-term care provision. | Nursing homes n=3  9 residents  9 medical professionals  9 management administrators  6 nursing home staff members  6 family members | NR/NR | End of life integrated care pathway / Dignity-Conserving End of life Care Model. | **Outcome measures:** McGill Quality of Life Questionnaire, Nursing Facilities Quality of Life Questionnaire, focus groups.  **Findings:** Although significant deterioration was recorded for physical quality of life, significant improvement was observed for social quality of life. Moreover, a clear trend toward significant improvements was identified for the quality of life domains of individuality and relationships. Three factors were required for the successful implementation of the intervention - regulatory empowerment, family centred care, and collective compassion. |
| Hockley and Kinley, 2016 (37)  United Kingdom  Intervention audit | To implement the Gold Standards Framework in Care Homes Programme and audit outcomes within nursing care homes across five clinical commissioning groups over a 7-year period using a research-based model of facilitation and to reflect on the practice development model. | Nursing homes n=76  NR | 2 years/NR | Gold Standards Framework in Care Homes | **Outcome measures:** Audit.  **Findings:** The percentage of residents dying in increased from 57% (19 NCHs) in 2007/8; to 79% (76 NCHs) in 2014/15 Further data revealed an increase in ACP (from 51% to 82%), the last days of life (from 21% to 60%) and cardio-pulmonary resuscitation decisions (from 52% to 87%). The percentage of residents dying in nursing care homes increased from 57% to 79%, with improvement in other outcomes. |
| Hockley et al, 2010, Watson et al, 2010 (38, 39)  United Kingdom  Evaluation - qualitative pre/post implementation | To report the impact of implementing both end of life care tools (Gold Standards Framework in Care Homes and an adapted LCP) together using the same facilitator while proactively visiting the nursing homes two to three times a month using a model of empowerment.  To reports on the qualitative interviews with bereaved relatives/friends and care home managers. | Nursing homes n=7  228 residents who had died  68 staff members  22 relatives pre implementation, 14 relatives/friends and 6 managers post implementation | 18 months / NR | Gold Standards Framework in Care Homes and an adapted LCP. | **Outcome measures:** Review of clinical notes of deceased, staff audit, qualitative interviews.  **Findings:** Do Not Attempt Resuscitation (DNAR) instructions rose by 72%. Written evidence of ACP conversations also increased from 4% to 53%. The use of the adapted LCP rose from 3% to 30% with three homes regularly using the documentation before the end of the project, 8% of staff returning the audit stated that the project had helped them realize the importance of ‘quality of life’ for residents rather than ‘striving to keep alive’. A third of people admitted they had only received end of life care training since taking part in the project. Over half said that the study had helped them prepare new staff for caring for dying residents and families, with staff from one of the nursing homes saying that this had never been done before. An apparent reduction in unnecessary hospital admissions and a reduction in hospital deaths from 15% deaths pre-study to 8% deaths post-study were also found. Post implementation, the results indicate more informed end of life decision-making involving families/friends, staff and GPs. |
| Horey et al, 2012 (40)  Australia  Action research/ evaluation | To investigate the acceptability and feasibility of using end of life care pathways in residential aged care facilities. | Residential aged care facilities n=14  63 residents  NR staff members | 14 months/NR | Introduction of end of life care pathways. | **Outcome measures:** Rate of pathway usage, interviews from staff members, hospital transfers, length of time on pathways, whether care was consistent with best practice.  **Findings:** Use of end of life care pathways across the facilities were in either low, moderate and high uptake groups - acceptability was critical to success implementation. There were fewer unnecessary admissions to hospital before death. The pathways encouraged documentation, and the audits demonstrated that care for residents on pathways was consistent with best practice of end of life. |
| in der Schmitten et al, 2014 (41) *  Germany  Controlled trial -evaluation | To evaluate the feasibility of implementing an Advanced Care Planning program specifically developed for use in German nursing homes and associated health care structures of a given town, and whether it leads to an increase in the number of clearly formulated, valid advance care plans. | Nursing homes n=13 (3 intervention and 10 in control)  575 residents | 16.5 months/  NR | Advanced Care Planning program; “beizeiten begleiten, based on the US “Respecting Choices” programme. | **Outcome measures:** Case notes, interviews with residents and the responsible nurse.  **Findings:** 49 (36.0%) participating residents completed a new advance directives over the period of the study, compared to 18 (4.1%) in the control region; these advance directives included 30 by proxy in the intervention region versus 10 in the control region. Proxies were designated in 94.7% versus 50.0% of cases, the advance directives was signed by a physician in 93.9% versus 16.7%, and an emergency order was included in 98.0% versus 44.4%. Resuscitation status was addressed in 95.9% versus 38.9% of cases. The implementation of an ACP program in German nursing homes led, much more frequently than previously reported, to the creation of advance directives with potential relevance to medical decision-making. |
| Kataoka-Yahiro et al 2017 (42)  USA  Evaluation | To evaluate a palliative and hospice care training of staff in two nursing homes in Hawaii - (a) to evaluate knowledge and confidence over three time periods, and (b) to compare staff and family caregiver satisfaction at end of program. | Long term care facilities n=2  52 staff members | NR/NR | Palliative and hospice care training palliative and hospice care training. | **Outcome measures:** Staff evaluation included knowledge and confidence surveys, pre- and post-test knowledge tests, and FAMCARE-2 satisfaction instrument.  **Findings:** The staff rated overall satisfaction of palliative care services lower than the family caregivers did. Statistically significant results were obtained for both self-rated perception of knowledge and confidence improvement in palliative and hospice care training (p<.05) The staff at 2 long-term care facilities who participated in the evaluation of palliative and hospice care training did very well in acquiring knowledge and confidence (p<.05), however, they expressed dissatisfaction of their performance in delivering palliative care services to their patients and families. |
| Kinley et al, 2014  Kinley et al, 2018 (43, 44)  United Kingdom  Cluster randomized controlled trial | To examine the impact of providing high facilitation and action learning when implementing the Gold Standards Framework for Care Homes programme. | Nursing homes n=38  1508 residents | 3 years/NR | Gold Standards Framework for Care Homes. | **Outcome measures:** Place of death, use of Integrated Care Pathway (ICP), undertaking ACP, having a cardiopulmonary resuscitation decision.  **Findings:** There were no significant effects in place of death. There was a significant effect in the high facilitation and action learning arm in the use of ICP. Undertaking ACP, having a cardiopulmonary resuscitation decision, revealed no significant effect. There was a significant association between the type of facilitation and the nursing homes completing the programme through to accreditation. Within the high facilitation and action learning arm, 83% (n=10/12) achieved accreditation compared to 27% (n= 3/11) in the high facilitation only arm (p=0.012). Within the observational group, 7% (n= 1/11) were externally accredited to have successfully implemented and embedded the programme into practice compared to 57% (n= 13/23) in the combined trial arms (p=0.005).  A greater proportion of residents died in those nursing homes receiving high facilitation and action learning but not significantly so. There was a significant association between the level of facilitation and nursing homes completing the programme through to accreditation. Year-on-year change occurred across all outcome measures. The nurse manager of a care home must be actively engaged when implementing the programme. |
| Kinley et al, 2017 (45)  United Kingdom  Programme implementation and audit, evaluation-audit | To describe the implementation of an end of life care programme to empower staff to meet their resident's end of life care needs. | Residential care homes n=71  118 staff members | 1 year/4 years | Steps to Success programme. | **Outcome measures:** Audit.  **Findings:** The audit found an increase of home deaths from 44% (n=8/18) within four residential care homes to 64% (n=74/115) in 23 residential care homes. There has been a corresponding increase in ACP discussions from 11% (n=2/18) to 58% (n=67/115) and completion of Do Not Attempt Cardiopulmonary Resuscitation (DNACPR) forms from 6% (n=1/18) to 63% (n=73/115). |
| Knight et al, 2008 (46)  United Kingdom  Evaluation | To give an overview of the education needs analysis carried out at the beginning of the project, whilst also exploring some of the methods through which the educational needs identified were addressed. | Nursing homes n=15  320 staff members | 3 years/NR | All Wales Integrated Care Pathway for the last days of life. | **Outcome measures:** Audit, questionnaire of knowledge to assess education needs.  **Findings:** The audit demonstrated an improvement in the recording of end of life care. The All-Wales Integrated Care Pathway use had increased from 3% to 31% in one year. |
| Kortes-Miller et al, 2007 (47)  Canada  Implementation and evaluation | To describe an approach to developing and delivering a research-based palliative care education curriculum in long-term care homes in rural north western Ontario that can serve as a model for other rural areas. The ultimate aim of providing palliative care education is to improve access to quality end of life care for seniors living in rural long-term care homes. | Long term care facilities n=3  128 registered nurses, registered practical nurses, health care aides and recreational therapists | 6 months / NR | The Palliative Care in Long Term Care curriculum. | **Outcome measures:** Participant evaluation (educational needs assessment survey).  **Findings:** Evaluations from every long-term care facility were very positive. Staff confidence and participation in delivering palliative care increased. |
| Kortes-Miller et al, 2015 (48)  Canada  Evaluation | To describe the development, implementation, and evaluation of a pilot educational intervention utilizing high fidelity simulation to improve unregulated care providers’ (frontline workers) confidence and skills communicating about death and dying in long term care homes. | Long term care facilities n=2  18 staff members | NR/NR | A high-fidelity simulation educational experience. | **Outcome measures:** Self- Efficacy in End of life Care survey, focus groups with unregulated providers, simulation lab education sessions, telephone interviews.  **Findings:** Quantitative data showed statistically significant improvements in participants’ self-efficacy scores related to communicating about death and dying and end of life care. Qualitative data indicated that the experience was a valuable learning opportunity and helped participants develop insights into their own values, beliefs, and fears providing end of life care. Pre–post results indicated statistically significant change in communication and patient management. Participants indicated they benefited by participating in the simulation through increased awareness, confidence, or comfort. |
| Kuhn and Forrest, 2012 (49)  USA  Pilot study/ evaluation | To evaluate a pilot program of palliative care education, training, consultations, and administrative coaching (pre training, 6-month post training and 12-month post training). | Nursing homes n=2  80 staff members  31 residents  33 family members | 1 year  NR | Palliative care intervention; including training, consultations and administrative coaching. | **Outcome measures:** Resident data from chart reviews, questionnaire of Palliative Care for Advanced Dementia.  **Findings:** Improvements were demonstrated on all measures (e.g. using 2 or more antipsychotics, laboratory draws, pain assessment, pain score, pain meds, antibiotic use, tube feeding, diet without restrictions, dietary supplements, body weight, hospital admissions, hospital referrals) for residents, staff members, and family members at site 2 but improvements were not demonstrated at site 1 except for pain assessment. |
| Lansdell and Mahoney, 2011 (50)  United Kingdom  Implementation study / evaluation | To drive up standards of end of life care in care homes to a best level of practice by providing a clearer, structured model for ongoing education and support. To develop a competency package that could be disseminated to other care homes. | Care homes n=4  NR | 3 years/NR | End of life care training programme (competency development package), including Principles of End of Life Care course. | **Outcome measures:** Self-reported feedback.  **Findings:** All of the feedback reported an increase in confidence with providing end of life care and in accepting appropriate specialist support. |
| Livingston et al, 2013 (51)  United Kingdom  Pre-test/post-test study / mixed methods study | To improve end of life care for people with dementia in a care home by increasing the number and implementation of advanced care wishes. | Nursing home n=1  98 residents  20 family members  58 staff members | NR/NR | End of life care intervention, including interactive training program. | **Outcome measures:** Family members interviewed after their relative died and completed quality of life, Quality of Life in Alzheimer’s Disease and General Health Questionnaire.  **Findings:** Post-intervention there were significant increases in documented advance care wishes arising from residents and relatives’ discussions with staff about end of life. These included do not resuscitate orders (16/22, 73% vs. 4/28, 14%; p<0.001); and dying in the care homes as opposed to hospital (22/29, 76% vs. 14/30, 47%; p<0.02). Bereaved relatives overall satisfaction increased from 7.5 (SD = 1.3) pre-intervention to 9.1 (SD = 2.4) post-intervention; p = 0.06. Relatives reported increased consultation and satisfaction about decisions. Staff members were more confident about end of life planning and implementing advanced wishes. |
| Lyon, 2007 (52)  Australia  Pre-post implementation study | To document implementation of best practice in ACP in a residential aged care facility using a cycle of audit, feedback and re-audit cycle audit with a clinical audit software program, the Practical Application of Clinical Evidence System. | Residential aged care facility n=1  46 resident files  14 staff members | NR/NR | Respecting Patient Choices. | **Outcome measures:** Documented evidence that the resident has been involved in ACP, that the residents family or significant others have had the opportunity to be involved in ACP, that staff who complete ACP have received training, have received regular education regarding end of life care issues, and there is evidence of ongoing assessment to ensure the ACP addresses all relevant issues as the resident's state of health alters.  **Findings**: The post-implementation audit showed a clear improvement as compliance ranged from 15-100% for the five audit criteria. |
| Magee et al, 2017 (53)  United Kingdom  Pre-test/post-test study / mixed methods study | To outline the process of introducing this programme into a care home and its impact upon those who were involved. | Care home n=1  5 staff members (registered nurses, care assistants and activity coordinator)  9 residents  3 residents' families | 4 weeks/NR | Namaste Care Programme. | **Outcome measures:** Cohen-Mansfield Agitation Inventory, the Cornell Scale for Depression in Dementia and the Challenging Behaviour Scale, focus groups with staff and family members.  **Findings:** The majority of participants had an improvement in all three of the scales Advantages from the programme included that staff found out details about the residents of which they had been previously unaware, having families involved in the delivery of the programme was helpful in terms of building relationships and it was easier to talk to them. |
| Mayrhofer et al, 2016 (54)  United Kingdom  Mixed method study / evaluation | The goal of the Train-the-Trainer pilot project was to consolidate the success of the ABC end of life care programme, increase the capacity of the care home workforce to provide end of life care, and develop a model that could sustain training in and provision of end of life care in care homes' and to identify what supported or hindered the uptake of the programme. | Care homes n=17  274 residents  34 staff members/trainers | 9 months/NR | Train-the-Trainer End of Life Care Education Programme. | **Outcome measures:** Service use logs, data collected using modified InterRAI forms, and care notes of residents who had died post intervention. Face to face interviews and focus groups.  **Findings:** Positive association between care home stability, in terms of leadership and staff turnover, and uptake of the programme. Care home ownership, type of care home, size of care home, previous training in end of life care and resident characteristics were not associated with programme completion. Working with facilitators was important to trainers, but insufficient to compensate for organisational turbulence. Variability of uptake was also linked to management support, programme fit with the trainers’ roles and responsibilities and their opportunities to work with staff on a daily basis. |
| McGlade et al, 2016 (55)  Ireland  Feasibility study | To identify challenges in implementing the ‘Let Me Decide’ ACP programme in long term care. | Nursing homes n=2  83 staff (senior nurses)  70 residents  NR family members | NR/NR | The ‘Let Me Decide’ - ACP programme. | **Outcome measures:** Residents who completed some form of end of life care plan, Standardised Mini Mental State Examination and Instrument to Assess Competency to Complete an Advance Directive.  **Findings:** Following implementation of the programme, more than 50% of residents in each of the three study sites had some form of end of life care plan in place. Of the 70 residents who died in the post-implementation period, 14% had no care plan, 10% (with capacity) completed an advance care directive and lacking such capacity, 76% had an end of life care plan completed for them by the medical team, following discussions with the resident (if able) and family. |
| Moore et al, 2017, Saini et al, 2016 (56) (57) *  United Kingdom  Feasibility study | To (1) understand how the intervention operated in nursing homes in different health economies; (2) collect preliminary outcome data and costs of an interdisciplinary care leader to facilitate the Intervention; (3) check the Intervention caused no harm.  To examine practices relating to end of life discussions with family members of people with advanced dementia residing in nursing homes and to explore strategies for improving practice. | Nursing homes n=2  9 residents  4 residents' family members  28 staff members/  19 staff interviews | 6 months/3 years | Compassion Intervention. | **Outcome measures:** Symptoms were recorded monthly for recruited residents. Semi structured interviews were conducted with nursing home staff, external healthcare professionals and family carers. Data collected on documented resuscitation status; a pain management plan; preferred place of death recorded; hospital admissions, emergency phone calls and location of deaths. Resident outcomes included Waterlow Scale (pressure ulcer risk), Neuropsychiatric Inventory, Cohen-Mansfield Agitation Inventory, Pain Assessment in Advanced Dementia Scale, Symptom Management at end of life in Dementia and Quality of Life in Late-Stage Dementia Scale. Carer outcomes included the Zarit Burden Interview, the Hospital Anxiety and Depression Scale, Satisfaction with Care at end of life in Dementia Scale and the Resource Utilization in Dementia Questionnaire.  Ethnography, fieldwork notes, observations recorded in a reflective diary and post-intervention in-depth interviews.  **Findings:** The intervention prompted improvements in ACP, pain management and person-centred care. Implementation was feasible to differing degrees across sites, dependent on context. The intervention provided insights into existing routines critical for driving practice improvements, often highlighting existing deficits in the care being provided.  Four major themes described strategies for improving practice: educating families and staff about dementia progression and end of life care; appreciating the greater value of in-depth end of life discussions compared with simple documentation of care preferences; providing time and space for sensitive discussions; and having an independent healthcare professional or team with responsibility for end of life discussions. |
| Morris and Galicia-Castillo, 2017 (58)  USA  Evaluation | To describe the Caring About Residents’ Experience and Symptoms (CARES) program, a model of palliative care for nursing home residents. | Nursing home n=1  170 residents | NR/NR | CARES Program. | **Outcome measures:** Symptom burden, treatment plans, goals of care hospitalizations.  **Findings:** Following consultation, 67% of residents had a change in code status. Of residents desiring a palliative course, 90% were never hospitalized. Overall, 53% of residents died; and those in long term care dying more often with hospice. |
| Nilsen et al, 2018 (59)  Sweden  Evaluation | To explore the organizational readiness to implement palliative care in nursing homes in Sweden based on the evidence-based guidelines to support staff. | Nursing home n=20  200 staff members / 20 nursing home managers | 6 months/NR | Educational intervention intended to facilitate the development of an evidence based palliative care. | **Outcome measures:** Interviews.  **Findings:** Analysis of the data yielded ten factors (i.e., sub-categories) acting as facilitators and/or barriers. Four factors constituted barriers: the staff’s beliefs in their capabilities to face dying residents, their attitudes to changes at work as well as the resources and time required. Five factors functioned as either facilitators or barriers because there was considerable variation with regard to the staff’s competence and confidence, motivation, and attitudes to work in general, as well as the managers’ plans and decisional latitude concerning efforts to develop evidence-based palliative care. Leadership was a facilitator to implementing evidence-based palliative care. |
| O’Brien et al, 2016 (60)  United Kingdom  Evaluation | An evaluation of the implementation of Six Steps with the first cohort of care homes to complete the programme; to explore the experiences of the facilitators of the programme, specifically with regard to the implementation approach they had adopted and to obtain a detailed account of the impact of Six Steps on individual care homes. | Care homes n=NR  18 CH staff- facilitators | 6 months/NR | Six Steps to Success programme. | **Outcome measures:** Questionnaire, case studies, interviews.  **Findings**: Post intervention improvement in ACP, improved staff communication/confidence when dealing with multi-disciplinary teams, improved end of life processes/documentation and increased staff confidence through acquisition of new knowledge and new processes. |
| Oliver et al, 2009 (61)  USA  Implementation study /  evaluation | To describe the experience of a yearlong quality improvement initiative using the Missouri Mortality Risk Index to facilitate discussions of goals of care for nursing home residents. | Nursing home n=1  NR | 12 months/NR | Missouri Mortality Risk Index to facilitate goals of care discussions. Predictive model based on the minimum dataset. | **Outcome measures:** Use of minimum dataset scores to identify residents for end of life care review, Cognitive Performance Scale and the Minimum Dataset-ADL Hierarchical Scale, Flacker score, Minimum Dataset Mortality Risk Index – Risk (MMRI-R) score.  **Findings:** The goals of the study were not met, however numerous challenges were identified (related to data generation/administration and logistics to implement the intervention). |
| Raunker and Timm, 2010 (62)  Denmark  Evaluation | To evaluate an attempt to develop-through three pedagogical methods-the palliative care competencies of the personnel and make organizational improvements at three Danish nursing homes. | Nursing homes n=3  22 staff members (assistant nurse, nursing aide, home helper, social worker) | 2 months/NR | Care initiative. | **Outcome measures:** Focus groups with nursing home staff members and teachers to review and evaluate the project and its benefits including reflection of their practice.  **Findings:** Staff felt that their competencies in palliative care had significantly improved and that the organizational initiatives taken had improved the palliative care efforts in the nursing home, although to a lesser degree. It highlights the need for recognition by colleagues, active involvement of nursing home managers, and a certain understanding of the methods, including the importance of prioritizing practice-based competence training. |
| Stacpoole et al, 2015, Stacpoole et al, 2017 (63, 64)  United Kingdom  Evaluation  Qualitative focus groups | To evaluate the effects of the Namaste Care programme on the behavioural symptoms of residents with advanced dementia in care homes and their pain management.  To establish whether the Namaste Care  program can be implemented in UK care homes; and what effect Namaste Care has on the quality of life of residents with advanced dementia, their families and staff. | Care homes n=2 /  n=6 (4 completed)  30 residents | 4 to 6 months/NR | Namaste Care Programme. | **Outcome measures:** Neuropsychiatric Inventory—Nursing Homes and Doloplus-2 behavioural pain assessment scale, Charlson index of co-morbidities, Bedford Alzheimer’s Nursing Severity Scale. Staff focus groups were held in each care home before and after the implementation of Namaste**.**  **Findings:** The severity of behavioural symptoms, pain and occupational disruptiveness decreased in four care homes. Increased severity of behavioural symptoms in one care home was probably related to poor pain management, reflected in increased pain scores, and disrupted leadership. Comparison of Neuropsychiatric Inventory—Nursing Homes scores showed that severity of behavioural symptoms and occupational disruptiveness were significantly lower after initiation of Namaste Care (n=34, p<0.001) and after the second interval (n=32, p<0.001 and p=0.003). However, comparison of these measures in the second and third intervals revealed that both were slightly increased in the third interval (n=24, p<0.001 and p= 0.001). The characteristics of care uncovered before Namaste was implemented were chaos and confusion, rushing around, lack of trust, and rewarding care. After the programme was implemented these perceptions were transformed, and themes of calmness, reaching out to each other, seeing the person, and, enhanced well-being, emerged. |
| Taylor and Randall, 2007 (65)  New Zealand  Evaluation | This article will consider and demonstrate the use of process mapping as a quality improvement tool to enhance the effective implementation and sustained use of the LCP for the dying patient within aged residential care. | Residential aged care facilities n=3  NR | 6 months/NR | LCP Pilot Project – including process mapping. | **Outcome measures:** Bottlenecks and solutions identified by members of the multidisciplinary team, pre-emptive prescribing.  **Findings:** The following bottlenecks were identified: GP not always available to prescribe for residents' symptoms as they occur, varying levels of GP knowledge and experience, GP may not have prescription pad or controlled drug prescribing pad when visiting the facility, unavailability of medications, access to syringe driver difficult in rural setting, access to equipment to assemble syringe driver, staff not deemed competent in use of syringe drivers, limited after-hours pharmacy services. Solutions to each bottleneck were also identified (e.g. pre-emptive prescribing increased post implementation for pain, agitation, respiratory tract and secretions, nausea and vomiting and dyspnoea). |
| Temkin-Greener et al, 2017a,  Temkin-Greener et al, 2017b (66, 67)  USA  Randomized controlled trial | To examine the efficacy of nursing home-based integrated palliative care teams in improving the quality of care processes and outcomes for residents at the end of life. | Nursing homes n=31  1018 staff members  NR residents | 2 years/NR | Improving Palliative Care through Teamwork (IMPACTT). | **Outcome measures:** Place of death, number of hospitalizations, self-reported pain and depression in the last 90-days of life, staff satisfaction surveys and impact on care processes and conducted rapid ethnographic assessments in all treatment homes using in-depth interviews.  **Findings:** Overall, no statistically significant effect of the intervention was found. However, independent analysis of the interview data found that only 6 of the 14 treatment facilities had continuously working palliative care teams throughout the study period. Decedents in homes with working teams had significant reductions in the odds of in-hospital death compared to the other treatment [odds ratio (OR), 0.400; p<0.001), control (OR, 0.482; p<0.05), and nonrandomized control nursing homes (0.581; p<0.01). Decedents in these nursing homes had reduced rates of depressive symptoms (OR, 0.191; p<0.01), but not pain or hospitalizations. |
| Unroe et al, 2015 (68)  USA  Evaluation | To reduce avoidable hospitalizations of long-stay residents using the Optimizing Patient Transfers, Impacting Medical Quality, and Improving Symptoms: Transforming Institutional Care (OPTIMISTIC) project. | Nursing homes n=19  910 residents | 12 months/NR | OPTIMISTIC Approach. | **Outcome measures:** Root-cause analyses for all acute transfers, structured interviews and physical examination with a focus on geriatric syndromes.  **Findings:** Of the transfers, 29% as avoidable (57% were not avoidable and 15% were missing), and opportunities for quality improvement were identified in 54% of transfers. Lessons learned in early implementation included defining new clinical roles, integrating into nursing facility culture, managing competing facility priorities, communicating with multiple stakeholders, and developing a system for collecting and managing data. |
| Verreault et al, 2018 (69)  Canada  Quasi-experimental study | To evaluate the impact of a multidimensional intervention to improve quality of care and quality of dying in advanced dementia in long-term care facilities. | Long term care facilities n=4  193 residents with advanced dementia and their close family members | 22 months/12 months | Multicomponent intervention, including training program clinical monitoring of pain, communication with families, and involvement of a nurse facilitator. | **Outcome measures:** Quality of care was assessed with the Family Perception of Care Scale. The Symptom Management for End of life Care in Dementia and the Comfort Assessment in Dying scales were used to assess the quality of dying.  **Findings:** The Family Perception of Care score was significantly higher in the intervention group than in the usual care group (157.3 vs 149.1; p = 0.04). The Comfort Assessment and Symptom Management scores were also significantly higher in the intervention group. The proportion of highly satisfied families was higher in the intervention group than in the control group (71.7% vs 55.3%), although the difference was not statistically significant. CAD-EOLD scores were significantly higher in the intervention group (35.8 vs 33.1, p= 0.03), and the difference remained statistically significant within all but one subscale. |
| Vis et al, 2016 (70)  Canada  Evaluation | To develop, implement, and assess the benefits of a peer-led debriefing intervention to help staff manage their grief and provide long term care homes an organizational approach to support them. | Long term care facilities n=4  23 staff members | 5 years/NR | The INNPUT intervention; a peer-led debriefing intervention to help staff manage their grief. | **Outcome measures:** Qualitative and quantitative questionnaires, field notes and interviews.  **Findings:** The intervention offered staff an opportunity to express grief in a safe context with others, an opportunity for closure and acknowledgment. |
| Waldron et al, 2008 (71)  United Kingdom  Evaluation | To describe an evaluation of a comprehensive palliative care education programme. | Nursing homes n=48  30 staff members (nurse manager, sister, staff nurse) | NR/NR | Palliative care education programme with link nurses (link nurse model) | **Outcome measures:** Survey of link nurses who attended the training course, including both qualitative and quantitative data.  **Findings:** 30/39 link nurses participated. The course and content was viewed positively, the link nurses felt they had benefited from the training course facilitator that the course material was good, and their knowledge and understanding increased. Many respondents (83%) had not commenced cascading training within their nursing home due to lack of time and competing mandatory demands. |
| Wickson-Griffiths et al, 2015 (72)  Canada  Semi structured individual /focus group interviews | To pilot evaluation of Comfort Care Rounds - a strategy for addressing long-term care home staff’s palliative and end of life care educational and support needs. | Long term care facilities n=2  40 participants | 18 months/10 months | Comfort Care Rounds Strategy. | **Outcome measures:** Semi structured individual and focus group interviews.  **Findings:** Study participants identified that effective advertising, interest, and assigning staff to attend Comfort Care Rounds facilitated their participation. The key barriers to their attendance included difficulty in balancing heavy workloads and scheduling logistics. Inter-professional team member representation was sought but was not consistent. |
| Wils et al, 2017 (73)  Belgium  Evaluation | To assess the effect of an education program for nurses on the registration of care goals in a nursing home with a population of elderly residents suffering from dementia, to explore the views of nursing home staff on ACP in patients with dementia. | Nursing home n=1  124 residents  13 nursing staff | 12 months/NR | Conceptual Framework for Implementation of ACP (model of care goals). | **Outcome measures:** Questionnaire on facilitating and obstructing factors concerning the implementation of ACP, pre-and post-measurement of all ACP-related registrations, based on a novel care goal model, semi-structured interviews.  **Findings:** Apart from the number of advanced directives (p=1.00) and appointed representatives (p=0.08), all items increased significantly in all residents still alive after the registration period p<0.05), intervention included all 124 residents diagnosed with dementia, including ACP conversation with appointed representative, ACP conversation with resident. Significant changes in caregiver’s views on ACP at the end of the intervention period. At 12 months, there were significant increase in the number of interviews regarding ACP held with the residents, and a significant increase in the number of care goals documented. |

*Additional information sourced from published protocol.

| **Supplementary material 3: Overview of implementation strategies identified** | | | | | |
| --- | --- | --- | --- | --- | --- |
| **Author / year**  **Country**  **Study design** | **Description of intervention** | **Facilitation** | **Details of training /education** | **Internal engagement** | **External engagement** |
| Aasmul et al, 2018 (1)    Norway  Cluster randomized controlled trial | Education programme to learn early and repeated communication with patients and families and to implement ACP. | **External facilitation:** Researchers were in contact with the nursing home units during the intervention period by means of regular telephone contact every second week to support the implementation.  **Internal facilitation:** Nurses attending the education seminar were named COSMOS ambassadors. After the two-day seminar, the COSMOS ambassadors were responsible for teaching their colleagues in the unit about the ACP process. The ambassadors were encouraged to find an optimal setting, according to their local routine, in which to train colleagues. Ambassadors were advised to talk during lunch and/or report (10-20 minutes) several times per week to enable optimal coverage. | Nursing home managers, registered and licensed practical nurses, and physicians related to the intervention group were invited to participate in a two-day education seminar, which offered a standardized education programme about ACP with patients and families. Nurses attending the education seminar were named COSMOS ambassadors. At least two nurses from each nursing unit, with hands-on experience with patients, were required to attend the education. It included lectures, skills training and role-play. | Nursing home managers registered and licensed practical nurses. | Physician, preferably with an established patient relationship, attended the quarterly meetings. |
| Agar et al 2017,  Luckett et al, 2017 (2, 3)  Australia  Cluster randomized controlled trial | Facilitated case conferencing organised by PCPCs. | **External facilitation:** NR  **Internal facilitation:** One registered nurse was trained as a PCPC in each nursing home working for two days per week or equivalent. Roles included identifying residents, organising case conferences, implementation of palliative care plans and training staff in palliative care. | Education sessions targeted at registered nurses. Training included integration of palliative care resources within care homes, such as establishment of a palliative care room, development of palliative care toolkits; and the introduction of an in-house palliative care team. | Care home staff (nursing, assistant director of nursing, manager, research nurses, allied health staff). | NR |
| Amador et al, 2016 (4)  United Kingdom  Mixed methods study | Appreciative inquiry (Social Identity Approach). | **External facilitation:** Facilitated by a palliative care nurse researcher, with experience in Appreciative Inquiry.  **Internal facilitation:** NR | Three appreciative inquiry sessions over a six-month period. | Care home staff - all staff members across the three homes. | Appreciative Inquiry meetings included care home staff and visiting health care practitioners, including GPs and district nurses. GPs and district nurses were invited to participate in the intervention. |
| Ampe et al, 2017 (5)  Belgium  Quasi-experimental pre-test/post-test study with an intervention and control group | ‘’We DECide’, an educational intervention for nursing home staff on shared decision‐making in the context of ACP for residents with dementia. | **External facilitation:** Each group was taught by an experienced communication trainer.  **Internal facilitation:** NR | Three modules, in the form of two 4 hour workshops and a homework assignment. Each module was designed to train the specific competences that are necessary to complete the corresponding step. | Care home staff - clinical staff, management. | NR |
| Andrews et al, 2009 (6)  Australia  Action research | Action research involved semi-structured interviews to staff, residents and family members, resulting in an information package provided to family caregivers. | **External facilitation:** Researcher acted as a facilitator.  **Internal facilitation:** NR | Ten meetings, consisting of two registered nurses, one enrolled nurse and two unregulated workers, and family members. | Care home staff - nurses, unregulated workers. | NR |
| Arcand et al, 2009 (7)  Canada  Pre-test/post-test study | Educational program and a booklet for staff, and optionally to families. | **External facilitation:** NR  **Internal facilitation:** A trained in-house geriatric clinical nurse specialist was designated to help organize the educational sessions and facilitate staff participation. | Educational sessions, lasting 45 minutes, for nursing staff. Physicians were similarly invited to attend a 60 minute session and given a relevant medical article for further reading. A 25-minutes phone interview to family members about the last week of care at least 3 months after the death of the resident. | Nursing/care home staff, head nurses.  Relatives | Consciousness raising meetings were organized with physicians, head nurses, other professionals, members of the residents' committee and administrators. Physicians were similarly invited to attend a 60 minute session and given a relevant medical article for further reading. |
| Badger et al, 2007, Badger et al, 2009, Badger et al, 2012 (8-10)  United Kingdom  Pre-test/post-test study and qualitative case study | Gold Standards Framework in Care Homes. | **External facilitation:** Care home managers were offered support over the 8 month period of introduction, by a local Gold Standards Framework in Care Homes facilitator and support by the development team, a helpline and conference calls.  **Internal facilitation:** NR | Four, one-day externally located workshops, delivered face to face. Following each workshop, staff implemented the programme in their care homes, supported by training materials and local Gold Standards Framework in Care Homes facilitators. | Nursing assistants, registered nurses, care home managers. | NR |
| Beck et al, 2012,  Beck et al, 2015 (11, 12)  Sweden  Quasi-experimental pre-test/post-test study and semi structured interviews | Circle sessions interspersed with workshops, semi-structured individual interviews. | **External facilitation:** One circle leader facilitated the study circle sessions and the workshops for the respective district. Three facilitators participated in a study circle leader course, a three-day workshop with two follow up days. All facilitators were employed in the respective district, two as registered nurses and one as a specialized, licensed practical nurse.  **Internal facilitation:** NR. | Seven, two-hour study circle sessions with three, six-hour workshops in between. The sessions included discussions and reflections of texts or tasks carried out prior to the meeting. A second circle group was formed consisting of all the managers and registered nurses working at the care homes and focused on how to support and guide the nurses. One or two nursing assistants from each study circle group and the manager and/or registered nurse at that facility took part in workshop sessions. The workshops focused on how practices could be changed in line with discussions. | Nursing assistants, registered nurses, managers. | NR |
| Blackford et al, 2007 (13)  Australia  Evaluation | Respecting Patient Choices. | **External facilitation:** Two full time project officers assisted the care homes to prepare for the implementation, including engagement of associated community/acute services in sector and engagement and support from GPs. Project officers also provided a two-day training course to selected care home staff to prepare nominated staff to be able to facilitate ACP discussions.with residents and families as well as complete appropriate documents related to ACP.  **Internal facilitation:** Facilitated discussions by selected care home staff, who attended a two day training to learn how to facilitate ACP discussions with residents and families as well as complete appropriate documents related to ACP. | A training course to selected staff. GPs were invited to an education session hosted by the local Division of General Practice and received a GP Information Kit on ACP. | Care home staff  Residents and family members. | Engagement of associated community/acute services in sector and engagement and support from GPs. GPs were invited to an education session as stated. |
| Booth et al, 2014 (14)  United Kingdom  Evaluation of educational interventions | Action learning project. | **External facilitation:** NR  **Internal facilitation:** The sessions were facilitated by the group themselves. | A 10 day action learning skills course, with a palliative care taught component typically lasted about 90 minutes each day. The education sessions included PowerPoint presentations, and educational DVD and Social Care TV. Students were given copies of each presentation and further reading, creating a large portfolio of resource material to cascade the learning in their own nursing home. | Managers or deputy managers. | NR |
|  | Six Steps to Success programme. | **External facilitation:** Programme delivered by one facilitator working three days per week over an eight month period. The programme materials were adapted by the facilitator to suit the locality and the needs of the care homes. Visits to the care homes between the sessions were also made for more intensive support in practice.  **Internal facilitation:** NR | Programme delivered in ten online study sessions or workshops over two whole days and eight half days. Two additional days of teaching explore key issues in more depth, including ACP. Each care home committed to delegating two attendees to the programme who were expected to attend every session when possible. Each care home was provided with a file containing comprehensive materials, which were added to over the course of the programme. | Care home staff. | NR |
|  | Gold Standards Framework for Care Homes. | **External facilitation:** Programme delivered through the end of life care coordinator team. Between workshops a support session was held in each locality, with homes from each area supported by their local end of life care coordinator.  **Internal facilitation:** NR | Training consist of an introductory workshop followed by four workshops to introduce care homes to end of life care standards and best practice. A final workshop looks at consolidation and accreditation. The programme was supported by an introductory DVD, a good practice guide, and a website. | Care home staff . | Collaboration with GPs and specialists. |
| Brajtman et al, 2012 (15)  Canada  Pilot study/evaluation | Educational intervention. | **External facilitation:** NR  **Internal facilitation:** NR | One module, delivered as self-directed learning about end of life delirium and inter professional practice. Each group participated in a one-hour session comprising a clinical encounter and received a didactic “theory burst” repeated two weeks later. | Care home staff. | NR |
| Brännström et al, 2005 (16)  Sweden  Evaluation | LCP. | **External facilitation:** The project principal investigator, the chief nurse for the municipality and the registered nurse responsible for care development held hour-long meetings every third month with the contact nurses to reflect on issues about end of life care.  **Internal facilitation:** Facilitated by a contact registered nurse appointed at each care home. Contact nurses completed a 35 hour, online train-the-trainer course. | One three-hour sessions on the intervention. Each contact nurse then taught staff at their respective workplace and acted as a resource person for LCP implementation.  A 2 x 3.5 hour course in end of life care for all staff working in residential care homes. | Nursing assistants, registered nurses, contact nurses. | Physicians included in the intervention. |
| Campion et al, 2016 (17)  United Kingdom  Implementation study | Education and training including clinical rounds, advice and guidance, communication, and care co-ordination. | **External facilitation:** Team included a palliative care consultant, a palliative care nurse consultant, a palliative care matron and three clinical nurse specialists. Care homes were facilitated by a clinical nurse specialist on the team, who undertake clinical rounds with the nursing home staff, once a month, with extra visits if necessary.  **Internal facilitation:** NR | Five, one-hour education sessions. The sessions are repeated in each nursing home until all staff have attended all sessions. Once they have completed all sessions, staff receive a certificate of attendance. The clinical nurse specialists undertake once a month a clinical round with the nursing home staff to help them identify residents who are approaching the terminal phase. Care homes received a resource folder which contained copies of the training sessions and further guidance. | Nursing home staff | Clinical rounds designed to coincide the GP or relative visit. |
| Chapman et al, 2018 (18)  Australia  Quasi-experimental design | Palliative Care Needs Round, including monthly onsite clinical meeting. | **External facilitation:** The intervention consisted of a new monthly onsite clinical meeting known as the Palliative Care Needs Round (referred to hereafter as ‘needs rounds’). These needs rounds were facilitated by a palliative care nurse practitioner.  **Internal facilitation:** NR | The intervention was developed to allow in-house training for the nurse practitioner in conducting the needs rounds. The needs rounds allowed for indirect specialist palliative care clinical input, staff education and support for residential facilities’ staff to prioritise residents for ongoing planning. | Facility staff | Ongoing planning discussions for residents (‘case conferences’) involved residents, their families, residential facility staff, the GP and the nurse practitioner as appropriate. |
| Chisholm et al, 2017, Hanson et al, 2016, Hanson et al, 2017 (19-21)  USA  Cluster randomized controlled trial  Evaluation | Two-component intervention: a video decision aid about goals of care choices and a structured decision-making discussion with the nursing home care plan team. | **External facilitation:** Research staff provided support to nursing home staff to promote the Goals of Care discussions during implementation.  **Internal facilitation:** Facilitated by a facility liaison from the care plan team at each nursing home. | One, one-hour training on the Goals of Care intervention including a printed discussion guide, and a role play of the discussion. | Care home staff | Physicians and nurse practitioners were invited to Goals of Care discussions. Family decision-makers were provided with a copy of the decision aid video and a print discussion guide. Family decision-makers were asked to participate in a care plan meeting with the care home interdisciplinary team. |
| Cornally et al, 2015 (22)  Ireland  Focus groups | ‘Let Me Decide’ - advance care-planning programme. | **External facilitation:** Support from the research team.  **Internal facilitation:** NR | Staff education on ACP considered focus groups and resources such as patient packs and laminated visual education aids. | Care home staff, clinical nurse managers and directors of nursing. | NR |
| Cox et al, 2017 (23)  United Kingdom  Exploratory mixed methods design with pre and post intervention evaluation | End of Life Care toolkit | **External facilitation:** End of life care toolkit designed and delivered by a clinical nurse specialist in palliative care, with support from a researcher and senior lecturer with expertise in communication skills training.  **Internal facilitation:** NR | Three training sessions of one hour each were delivered within each care home: an introduction to the toolkit, and a session on compassion; a session on communication and end of life care; and a session considering end of life symptoms. Eighteen training sessions were conducted within the six care homes during a 3 month period. | Care home staff. | The toolkit was designed with an expert steering group (two doctors working in local hospices, two geriatricians, and an academic specialising in cancer and palliative care). |
| Cronfalk et al, 2015 (24)  Sweden  Focus groups | Three competence-building programs between specialized palliative care units engaged by the county councils and nursing homes. | **External facilitation:** NR  **Internal facilitation:** NR | Education of 1–2 key persons per ward, including three, two-hour seminars, introducing the principles of palliative care. The program introduced role-play as a pedagogical means for learning. | Registered nurses, enrolled nurses, care assistants. | NR |
|  |  | **External facilitation:** Seminars followed by a consultation and support visits once a month for a year.  **Internal facilitation:** NR | Separate seminars for staff (5 x 2 hours for enrolled nurses and care assistants, and 4 x 2 hours for registered nurses), with a focus on the principles of palliative care and the palliative team, followed by consultation and support visits once a month for a year. | Registered nurses, enrolled nurses, care assistants. | NR |
|  |  | **External facilitation:** NR  **Internal facilitation:** NR | Three shared seminars (about 1.5 hours) introducing the LCP. | Registered nurses, enrolled nurses, care assistants. | NR |
| Farrington, 2014 (25)  United Kingdom  Pre-test/post-test study and semi structured interviews | The ‘ABC’ course, a blended e-learning programme (face-to-face facilitated workshops alongside online content). | **External facilitation:** Two nurses with significant experience in end of life care delivery and training acted as facilitators. Facilitators led the workshops and meetings, and were available to participants for additional contact via email both during and after the course.  **Internal facilitation:** NR | Blended learning component, including six one-hour online modules and followed by five facilitated workshops. The workshops took the format of facilitated discussion led by the ABC course facilitators with reference to online materials, and lasted between 60 and 90 minutes. | Care home staff | NR |
| Fernandes, 2008 (26)  Australia  Pre-test/post-test study | The Getting Research into Practice (GRIP) process of the Practical Application of Clinical Evidence System program. | **External facilitation:** Activities facilitated by the study author.  **Internal facilitation:** NR | Formal and informal in service, one to one education sessions. Education was provided on different topics related to ACP and management of end of life care. Residents and their relatives were invited to attend one of two separate sessions held on different days at different times. Additionally, residents and nursing staff held ‘one on one’ meetings with residents/relatives where the advance care plan was the main topic of discussion. Multidisciplinary meetings with a specific emphasis on an advance care plan were held twice weekly with residents and their relatives. | Managers and general staff | Multidisciplinary meetings held twice weekly, included resident’s GP, the director of nursing, the deputy director of nursing, nurse educator, physiotherapist, lifestyle coordinator and the registered nurse on duty. |
| Finucane et al, 2013 (27)  United Kingdom  Evaluation | Gold Standards Framework in Care Homes. | **External facilitation:** Facilitated by two community palliative care clinical nurse specialists who each spent one day per week working with care home managers, staff and GPs and provided education. Support by phone/in person outside of these meetings as required. Care home staff were encouraged to implement anticipatory care plans from admission. They were trained to use the adapted LCP when residents were identified as approaching death.  **Internal facilitation:** NR | Education programme based on the Macmillan Foundations in Palliative Care for Care Homes, provided by facilitator. Staff across all care homes were invited to attend each workshop, and the number of workshops was based on staff need and clinical nurse specialist time. Each workshop lasted 2.5 hours and was facilitated by both nurse specialists. Nine care home staff shadowed a nurse specialist and hospice staff for a day. | Care home staff - care home managers, staff. | Multidisciplinary team meetings, including the nurse specialist and GP. |
| Frey et al, 2017 (28)  New Zealand  Pre-test/post-test study and interviews | Supportive Hospice Aged Residential Exchange. | **External facilitation:** Clinical coaching by a specialist palliative care nurse through direct (for complex needs) and indirect (not so complex needs) patient consultation.  **Internal facilitation:** NR | Training included clinical coaching by a specialist palliative care nurse through patient consultation, role modelling of ACP conversations and debriefing amongst all staff following a resident's death. | Care home staff – nurses. | ACP conversations and debriefing following a resident's death with GPs. |
| Garden et al, 2016 (29)  United Kingdom  Evaluation | Bromhead Care Home Service - Education programme based on the Stop Delirium! Material. | **External facilitation:** Two registered general nurses with extensive experience in care of inpatients with dementia and frailty were seconded to provide for a two-year period, supported by a consultant liaison psychiatrist.  **Internal facilitation:** NR | An education programme was developed based on the Stop Delirium! material delivered via small group teaching 6–8 times in each care home to ensure all members of staff had participated. Educational material was developed on eating, drinking and dysphagia. Care homes were given a reference file with the information. | Care home staff | Facilitation supported by a consultant liaison psychiatrist, GPs asked to refer residents to service is approached, and endorse advance care plans. |
| Giuffrida, 2015 (30)  USA  Evaluation | Comprehensive palliative care program. | **External facilitation:** NR  **Internal facilitation:** NR | Included educating staff about goals of care, educating residents and families about palliative care philosophy, discussions of palliative care in daily morning report on residents whose health was declining. | NR | Establishment of regular meetings of the palliative care committee. |
| Hall et al, 2011 (31)  United Kingdom  Evaluation - qualitative methods | Gold Standards Framework for Care Homes. | **External facilitation:** NR  **Internal facilitation:** NR | Curriculum includes resources, learning aids and tools with adaptations to meet the needs of local areas. Most staff described some training in end of life care. The extent and type of training varied considerably between homes. | NR | NR |
| Hasson et al, 2008 (32)  United Kingdom  Evaluation - qualitative study | Palliative care educational programme and link nurse role. | **External facilitation:** An independent clinical practitioner, specializing in palliative care, delivered initial education to nursing home staff, and provided support to link nurses within each home. A full-time nurse co-coordinator prepared and assisted volunteer link nurses in delivering the educational programme.  **Internal facilitation:** Link nurses, who disseminated information from the coordinator into each nursing home. Monthly meetings were held with the facilitator and link nurses to reassess educational needs. | Educational programme, provided to nursing home staff, of facilitated learning for care home staff including expert opinion, a review of the literature, educational courses and the Macmillan Foundations in Palliative Care learning pack. A link nurse assisted in delivering an educational programme consisting of an information pack. Each link nurse was provided with a resource file outlining the palliative care educational programme for registered nurses and other care staff. This also gave details of hospices and other services which nursing homes could access for support and advice. | Care home staff - link nurses. | NR |
| Hewison et al, 2008 (33)  United Kingdom  Case study approach /  evaluation - qualitative study | Gold Standards Framework for Care Homes. | **External facilitation:** Facilitators were nurses or GPs who had experience of using the Gold Standards Framework.  **Internal facilitation:** A Gold Standards Framework coordinator was identified from the home staff to act as a link between the external facilitator and staff. | A one-day launch event followed by facilitators working with small groups of care homes to assist them with the Gold Standards Framework, followed by three one-day workshops. | Care home staff. | NR |
| Hickman et al, 2016 (34)  USA  Implementation study | Advanced Care Planning, using a structured interview guide. | **External facilitation:** Facilitated by a full-time specialized palliative care registered nurse developed for the project. Facilitators received training including the End of life Nursing Education Consortium geriatric curriculum, a comprehensive palliative care educational program.  Facilitators were supported by six nurse practitioners and a team of geriatricians. Facilitators were trained using an online training module, followed by 8.5 hours of face to face role playing and education. Facilitators also completed additional role-playing activities, and some were trained as certified instructors.  **Internal facilitation:** NR | Facilitators provided in service training to nursing home clinical staff. Training included the End of life Nursing Education Consortium geriatric curriculum and the Respecting Choices Last Steps intervention. The program requires independent online training modules followed by 8.5 hours of face-to-face role-playing and education. Educational handouts on selected topics were also used to guide conversations and support informed decision-making. Educational sessions were offered to residents and families. | Registered nurses.  Residents and relatives |  |
| Ho et al, 2016a,  Ho et al, 2016b (35, 36)  China  Evaluation | End of life integrated care pathway / Dignity-Conserving End of life Care Model. | **External facilitation:** Facilitated by an interdisciplinary end of life care team consisting of three core members with expertise in social work, nursing and medicine. The end of life care team was shared between all three nursing homes. A project officer with background in palliative nursing was responsible for providing nursing care to all program participants and delivering training to other nursing staff.  **Internal facilitation:** The trained staff were encouraged to cascade this training down. | Two module training programs combined to impart an overarching philosophy of holistic care in practice. An annual fieldwork attachment program with overseas palliative care training institutes was developed and provided to managerial staff and senior care professionals of each nursing home. The skills and knowledge obtained were transferred to all formal care workers through a train-the-trainer paradigm. Education talks and seminars were offered to interested residents and their families | Care home staff, nurses, social workers, personal care workers. | An interagency care co-ordination protocol was established with two partnering hospitals to provide acute and convalescent care as well as medical advice and support for terminally ill residents. |
| Hockley and Kinley, 2016 (37)  United Kingdom  Intervention audit | Gold Standards Framework in Care Homes. | **External facilitation:** The programme was facilitated by the Care Home Project team. The nurse facilitators visited the care homes every 7–10 days to establish good relationships with staff/ management and to role model aspects of the programme.  **Internal facilitation:** Each care home was encouraged to appoint at least two coordinators who would lead the implementation. During the pre-implementation period, these coordinators attended the Foundations in Palliative Care for Care Homes course held in local care homes. | Four workshops. Additional training included an ‘Introduction to palliative care day’ for all new staff, the Macmillan Foundations in Palliative Care (4 days over 2 months) course for carers and nurses, and action learning sets attended by managers every 2–3 months. | Care home staff | Each care home arranged for meetings where staff, external healthcare professionals (e.g. GP) and families were informed about the programme. |
| Hockley et al, 2010, Watson et al, 2010 (38, 39)  United Kingdom  Evaluation - qualitative pre/post implementation | Gold Standards Framework in Care Homes and an adapted LCP. | **External facilitation:** Facilitated by an experienced palliative care nurse, who visited each care home every 10–14 days. The nature of the contact included attending monthly register meetings alongside GPs; scenario-based teaching on death, and regular meetings with management/champions, role modelling good palliative care as the opportunity arose and facilitating debriefing sessions following a death.  **Internal facilitation:** Two key champions were appointed in each home and were responsible for co-ordinating and embedding changes. Key champions attended four workshops over the year. Key champions attended a four day facilitative learning course ‘Foundations in Palliative Care for Care Homes’ and were encouraged to cascade this training down to their own staff with the help of the facilitator. Champions implemented two main systems: The Gold Standards Framework in Care Homes 'supportive/palliative care register' or the 'adapted LCP'. | One, two-hour scenario based training. All staff were encouraged to attend 2-hour scenario-based training where they practised using the LCP documentation. Each manager organized the training over a two week period. | Care home staff -managers, key champions, all staff. | GPs were invited to attend these monthly meetings alongside the facilitator. |
| Horey et al, 2012 (40)  Australia  Action research/ evaluation | Introduction of end of life care pathways. | **External facilitation:** NR  **Internal facilitation:** NR | NR | Care home staff, managers. | Care pathways involved GPs |
| in der Schmitten et al, 2014 (41)  Germany  controlled trial -evaluation | Advanced Care Planning program; “beizeiten begleiten, based on the US “Respecting Choices” programme. | **External facilitation:** The research team attended a one-week training course to become certified facilitators and instructors for Respecting Choices. They developed an ACP program tailored to the German nursing homes.  **Internal facilitation:** Facilitated by two to four non-physician facilitators from each care home. | One, 20-hour training course for the two to four facilitators from each care home. Physicians received four hours of training. Education sessions were provided to nursing staff at the care home, nursing staff at the regional hospital; medical and paramedic emergency staff and professional guardians. | Care home staff. | Physicians were offered four optional 1.5-hour meetings over 2 years. Separate information events for nursing staff at care homes and at the regional hospital, for hospital and emergency physicians, for emergency medical service paramedical staff, and for professional guardians. |
| Kataoka-Yahiro et al 2017 (42)  USA  Evaluation | Palliative and hospice care training palliative and hospice care training. | **External facilitation:** NR  **Internal facilitation:** NR | One four hour communication skills workshop and a ten week culturally appropriate palliative and hospice care training. An interactive communication workshop followed the modular sessions that involved lecture and small group sessions. Each session accommodated a majority of employees working on day and evening shifts. Sessions were videotaped for those who were not able to attend the sessions. | Care home staff. | Hospital staff who attended the palliative and hospice care training included both experienced clinical staff from various disciplines and nonclinical staff (administration and education). |
| Kinley et al, 2014  Kinley et al, 2018 (43, 44)  United Kingdom  Cluster randomized controlled trial | Gold Standards Framework for Care Homes. | **External facilitation:** A facilitator visited nursing homes two to three times a month along with attendance at four GSFCH workshops. The facilitator helped coordinators to implement the Liverpool Care Pathway (LCP)/integrated care pathway (ICP), providing ongoing induction days for new staff and ongoing training.  **Internal facilitation:** Two coordinators were appointed from each nursing home. Coordinators attended a 4-day training on the Macmillan ‘Foundations in Palliative Care for Care Homes’ curriculum. | In the high facilitation and action-learning arm of the study, each nurse manager was asked to attended one, three-hour action learning set every month between the first and fourth Gold Standards Framework for Care Homes workshops. Action learning centred on 'leadership' in relation to implementing the framework programme. | Care home staff, care home managers. | NR |
| Kinley et al, 2017 (45)  United Kingdom  Programme implementation and audit, evaluation-audit | Steps to Success programme. | **External facilitation:** Facilitator visited care homes at least twice a month to help implement the programme and role model discussions and care where required.  **Internal facilitation:** NR | One, four-day Macmillan Foundations in Palliative Care for care homes training provided to the care home manager and senior carers. Training consisted of half-day seminars and action learning sets. An introduction to palliative care day was run monthly for all new members of staff to the care home. | Care home staff, care home managers. | GPs, district nurses and specialist palliative care teams informed about study. Managers encouraged to attend multidisciplinary team meetings with a GP. |
| Knight et al, 2008 (46)  United Kingdom  Evaluation | All Wales Integrated Care Pathway for the last days of life. | **External facilitation:** The project coordinator, a senior nurse, facilitated and funded the study days, in collaboration with the local specialist palliative care teams from across South-East Wales.  **Internal facilitation:** The coordinator set up a learning contract with the link senior nurse in individual care homes (usually the matron) which outlined expectations around times, numbers of nurses, participation, venue and various other ground rules prior to providing education. | Education included a standardized Integrated Care Pathway education pack, formal and informal teaching sessions and afternoons, a ‘Train the Trainer’ syringe driver training implemented over a two-year period, informal training and support sessions, including post-death debriefing sessions, study days, covering issues around improving end of life care. | Care home staff – nurses. | NR |
| Kortes-Miller et al, 2007 (47)  Canada  Implementation and evaluation | The Palliative Care in Long Term Care curriculum. | **External facilitation:** Facilitators were recognized palliative care providers from the community who had received palliative care education through a train-the-trainer program. Facilitators received an introduction to the course by the course curriculum developer, and offered face-to-face meetings, and telephone or email support throughout the course delivery.  **Internal facilitation:** A recognized leader within the facility, who had expertise in palliative care, act as a facilitator of the education. | Six, 2.5 hour training sessions. Care home staff received PowerPoint slides for each session, group exercise materials, case study exercises, and a list of palliative care resources on the topics covered. The facilitator's package contains additional reading material and resources on each topic, ice breakers, suggestions for group interaction exercises, case studies, suggested questions for group discussions, and a list of available educational videotapes. | Care home staff. | NR |
| Kortes-Miller et al, 2016 (48)  Canada  Evaluation | A high-fidelity simulation educational experience. | **External facilitation:** A high fidelity simulation educational experience was facilitated by two of the researchers on two separate occasions.  **Internal facilitation:** Two unregulated providers working as research collaborators informed their peers of the simulation learning opportunity and promoted their perception of the benefits. | Two, high fidelity simulation educational experiences lasting 3.5 hours. A participant manual was given to the staff. | Unregulated care providers. | NR |
| Kuhn and Forrest, 2012 (49)  USA  Pilot study/ evaluation | Palliative care intervention; including training, consultations and administrative coaching. | **External facilitation:** Facilitated by a project nurse with experience and expertise in dementia and palliative care, who provided weekly and as needed support for nurses and nursing assistants.  **Internal facilitation:** NR | Training consisted of 12 hours of interactive sessions at each nursing home, delivered in six modules at times convenient for all shifts, including education booklet provided to all participating family members and staff members. Case consultations by a project nurse with experience and expertise in dementia and palliative care were provided weekly and as needed for nurses and nursing assistants. The administrative coaching component consisted of monthly meetings of ‘‘Comfort Care Advisory’’ committees established at each nursing home. | Care home staff  Relatives | One palliative care consultation by physicians from the hospice for all enrolled residents and their available family members. A contractual relationship was formed with a local non-profit hospice to assist with training and palliative care consultations. |
| Lansdell and Mahoney, 2011 (50)  United Kingdom  Implementation study / evaluation | End of life care training programme (competency development package), including Principles of End of Life Care course. | **External facilitation:** Facilitated by staff from a local hospice who delivered a competency assessment training day.  **Internal facilitation:** Key staff from each care home who participated in the training disseminated the information, through team meetings and through supervision. | Ten, fortnightly one hour sessions with participants from each care home to introduce the study. A five day education course, principles of end of life care. | Care home staff.  Relatives | Relationship building between local hospice and care home. |
| Livingston et al, 2013 (51)  United Kingdom  Pre-test/post-test study / mixed methods study | End of life care intervention, including interactive training program. | **External facilitation:** Facilitated by a consultant physician and care home senior managers, who were part of the research team.  **Internal facilitation:** NR | Ten sessions of a manualized interactive training program. | Care home staff - general nurses, residential and senior care workers. | NR |
| Lyon, 2007 (52)  Australia  Pre-post implementation study | Respecting Patient Choices. | **External facilitation:** External assistance was provided by the Manningham Centre, who provided training related to the Respecting Patient Choices Program.  **Internal facilitation:** The facility's Palliative Care Best Practice Group supported the project. | Training included a total of 16 contact hours and additional one-to-one assistance with an experienced mentor when holding discussions with residents. Information sessions on ACP were conducted for nursing and medical staff, residents and their families. | Care home staff - general nurses. | Monthly meetings of the Palliative Care Best Practice Group, including GPs and other health professionals. Information kits were prepared for the doctors who did not attend the session. |
| Magee et al, 2017 (53)  United Kingdom  Pre-test/post-test study / mixed methods study | Namaste Care Programme. | **External facilitation:** The facilitator held a training session for staff and carers to be involved with the programme prior to its commencement. The first session of the programme was delivered by facilitator but was then run by the activity therapist with help from relatives.  **Internal facilitation:** NR | One two-hour session to participate in Namaste Care Programme activities with participation of residents, staff and family carers. | Care home staff, managers.  Residents and relatives | NR |
| Mayrhofer et al, 2016 (54)  United Kingdom  Mixed method study / evaluation | Train-the-Trainer End of Life Care Education Programme. | **External facilitation:** Facilitated by End of Life Care Educators/ facilitators who held various roles including palliative link nurse, palliative care nurse, practice-development nurses for care homes, end of life care specialist and end of life educator. All trainers had completed the ABC training.  **Internal facilitation:** The project trained two ‘trainers’ per care home, who subsequently trained six ‘learners’ each. Trainers’ responsibilities included the preparation of on-line and face-to-face teaching sessions, the organisation and facilitation of group discussions, and offering learners bite-size micro-teach sessions in daily practice. | Trainers took six end of life care training modules and three skills training workshops to support their trainer role, including input pertaining to learning and teaching methods, and practice workshops with educators/facilitators. The study aimed to train two ‘trainers’ per care home, who in turn were to train six ‘learners’ each. | Care home staff. | NR |
| McGlade et al, 2016 (55)  Ireland  Feasibility study | The ‘Let Me Decide’ - ACP programme. | **External facilitation:** Staff were supported by the research team. During monthly feedback meetings, any issues arising during implementation were discussed and changes were made to the programme to address the needs identified.  **Internal facilitation:** NR | Two half-day workshops covering the principles of palliative care, communication skills, bereavement and symptom assessment and management, were delivered to nurses and healthcare assistants. | Care home staff. | NR |
| Moore et al, 2017 Saini et al, 2016 (56)  (57)  United Kingdom  Feasibility study | Compassion Intervention. | **External facilitation:** The intervention was facilitated by an interdisciplinary care leader, employed full time to work in two nursing homes for six months. The interdisciplinary care leader was present in each care home for three half-days per week and with two nursing homes to provide mentoring, role modelling, advice and training.  **Internal facilitation:** Facilitated by two key champions, appointed in each care home. Facilitators were responsible for co-ordinating and embedding changes, and encouraged to cascade this training down to their own staff with the help of the external facilitator. | Formal staff and family training sessions ran by the facilitator, including informal on the job advice and support. | Care home staff, managers. | Weekly core team meetings, including the clinician, care home nursing staff and the interdisciplinary care leader. Monthly wider team meetings consist of the core team plus any local health and social care professionals and specialists, including GPs. |
| Morris and Galicia-Castillo, 2017 (58)  USA  Evaluation | CARES Program. | **External facilitation:** NR  **Internal facilitation:** NR | One, one hour in-service and online training by the palliative care physicians provided on: basics of palliative care, goals of care, pain, comprehensive assessment of non-pain symptoms, end of life care, and bereavement/self-care. | Care home staff. |  |
| Nilsen et al, 2018 (59)  Sweden  Evaluation | Educational intervention intended to facilitate the development of an evidence based palliative care. | **External facilitation:** The seminars were led by five registered nurses and researchers and one registered nurse who worked clinically, all with experience from working as nurses in palliative and geriatric care settings. Facilitators provided flexible support to homes, which was individually tailored to the needs of each home, consisting of a one to one visit to each home between each step to provide support. All facilitators also provided additional support and documentation via telephone and email throughout the programme.  **Internal facilitation:** The participants were selected by the manager of each nursing home, to continue as seminar leaders for further training of the entire staff at each nursing home. | The educational intervention consisted of five seminars. The seminars combined lecture style presentations and more interactive group discussions. They were provided as an outreach course and took place within nursing homes.  The research team developed an educational booklet primarily based on the two knowledge documents, including recommended assignments to do as preparations before each seminar and assignments to complete after each seminar. A list of references for further self-studying was also given in the booklet. The seminar group at each nursing home consisted of 8–10 participants and met approximately once a month over a period of 6 months. | Care home manager, assistant nurses, registered nurses. | The seminar content was determined after the discussions with staff, informal caregivers, and patients representing both hospital and community care. |
| O’Brien et al, 2016 (60)  United Kingdom  Evaluation | Six Steps to Success programme. | **External facilitation:** A facilitator delivered the workshops, and provided guidance and continual support to the implementation of end of life care changes in the home.  **Internal facilitation:** Nominated care home staff, champions, led the Six Steps programme, they attended the workshops and cascade the information to all home staff. | A workshop format addressing the core phases of end of life care within a six-stage cycle. | Nurses. | Occupational therapists and physiotherapists involved. |
| Oliver et al, 2009 (61)  USA  Implementation study /  evaluation | Missouri Mortality Risk Index to facilitate goals of care discussions. Predictive model based on the minimum dataset. | **External facilitation:** NR  **Internal facilitation:** NR | NR | Care home staff. | Physicians and facility social worker were involved. |
| Raunker and Timm, 2010 (62)  Denmark  Evaluation | Care initiative. | **External facilitation:** Teachers employed at a local university.  **Internal facilitation:** NR | Training was provided based on a selection of topics as requested by the care home, including clinical knowledge about death, communication, law and ethics and multi professional cooperation, attitudes towards life and death, clinical guidelines, everyday aesthetics. | Care home staff. | Social workers and physicians involved. |
| Stacpoole et al, 2015, Stacpoole et al, 2017 (63, 64)  United Kingdom  Evaluation  Qualitative focus groups | Namaste Care Programme. | **External facilitation:** Researchers acted as external facilitators. Following the workshop, the researchers visited each care home for a day, within the same week as the training, holding 20-minutes ‘teaching huddles’ explaining Namaste to as many staff as possible. A further visit the following week included role modelling a Namaste session.  **Internal facilitation:** One care worker was allocated responsibility for up to eight residents with advanced dementia in the Namaste space, while others take responsibility for the remaining residents. The Namaste care workers were chosen by their managers because they commanded respect within the care team, based on seniority and/or personality. | A one-day workshop attended by each care home manager and at least two designated Namaste Care workers from each care home. The workshop included teaching about advanced dementia, end of life care, and outlining the theory and practice of Namaste. Each manager received two copies of a book on Namaste and information about their role in the research. | Care home staff. | Meetings with family/friends of a relative |
| Taylor and Randall, 2007 (65)  New Zealand  Evaluation | LCP Pilot Project – including process mapping. | **External facilitation:** A LCP facilitator led the process mapping meetings and took responsibility for ensuring that solutions were actioned.  **Internal facilitation:** NR | An intensive education programme explaining the use of the LCP in practice was offered to clinical staff. A process mapping meeting was arranged at each facility with members of the interdisciplinary team. | Manager, care manager, senior nurse, local pharmacist. | Local pharmacist |
| Temkin-Greener et al, 2017a,  Temkin-Greener et al, 2017b (66, 67)  USA  Randomized controlled trial | Improving Palliative Care through Teamwork (IMPACTT). | **External facilitation:** A TeamSTEPPS master worked within the team in each facility. Facilitated by a study nurse interventionist, a geriatric nurse practitioner certified in End of life Nursing Education with significant nursing home practice experience. During the passive phase, the nurse interventionist was available to further coach the team on as needed/requested basis.  **Internal facilitation:** NR | Two training-education intervention components:  1. TeamSTEPPS (Strategies & Tools to Enhance Performance and Patient Safety), used to develop palliative care teams.  2. End of Life Nursing Education (ELNEC) - six, one-hour training modules provided ion the facility to all palliative care team members and to all direct care staff. Workshops were taught by the study nurse interventionist. Once staff completed ELNEC training, their facility was provided free on-line access to online modules for a three-year period. | Registered and licensed practising nurses, certified nurse assistants. | Physician’s assistants and physicians involved in the development of palliative care teams. Social workers and therapists involved in the intervention. |
| Unroe et al, 2015 (68)  USA  Evaluation | Optimising Patient Transfers, Impacting Medical Quality, and Improving Symptoms: Transforming Institutional Care (OPTIMISTIC) approach. | **External facilitation:** Facilitated by OPTIMISTIC nurses; seven full-time employed nurse practitioners covered three to four facilities each, coordinated with the internal registered nurses, and complemented the care of primary care providers by providing in-person evaluation and management of residents with acute changes or recent transition from the hospital.  **Internal facilitation:** Full-time registered nurses at each nursing facility lead the intervention addressing changes in condition and leading quality improvement efforts. | Training for OPTIMISTIC staff was a 2-week “boot camp” designed to introduce them to the overall project. OPTIMISTIC staff were trained in the Respecting Choices Last Steps Staff also receive the ELNEC curriculum, a train-the-trainer educational program designed to improve palliative care in the long-term care setting. | Registered nurses, facility staff. | Physicians involved in each collaborative care review. |
| Verreault et al, 2018 (69)  Canada  Quasi-experimental study | Multicomponent intervention, including training program clinical monitoring of pain, communication with families, and involvement of a nurse facilitator. | **External facilitation:** A local committee composed of an administrator, head nurses, and a physician was formed to facilitate the intervention.  **Internal facilitation:** The two local nurse facilitators in the intervention facilities were selected among the regular staff for their interest in end of life care and leadership with colleagues. The facilitators were released from their regular responsibilities for one year in order to work exclusively for the project. They received a 35 hour training in palliative care in advanced dementia. Facilitators helped researchers in organizing the training sessions, provided training to care home staff, acted as a coach for the nursing staff and facilitated communication between nurses, physicians, and family members. | Staff training sessions were completed followed by continuous involvement of a nurse facilitator in the intervention settings (seven hours for nurses and 3.5 hours for nurses’ aids). The nurse facilitators trained the nursing staff to use a pain assessment. Three hour training offered to physicians. | Care home staff. | Facilitators provided written information in the form of a booklet entitled Comfort Care at the end of life for persons and organized a meeting between the family member and the physician. |
| Vis et al, 2016 (70)  Canada  Evaluation | The INNPUT intervention; a peer-led debriefing intervention to help staff manage their grief. | **External facilitation:** Researchers trained unregulated care providers to become facilitators.  **Internal facilitation:** Volunteer unregulated care providers became facilitators. | Two training sessions provided on disenfranchised grief. | Care home staff/ unregulated care providers (front-line staff) - personal support workers, health care aids, nurse´s aids, nursing assistants. | NR |
| Waldron et al, 2008 (71)  United Kingdom  Evaluation | Palliative care education programme with link nurses (link nurse model). | **External facilitation:** A palliative care education facilitator coordinated and delivered the training.  **Internal facilitation:** Trained link nurses delivered the training provided by the external facilitator to other staff. | Education on the “Foundations in Palliative Care” delivered in-house to link nurses, in central venues using a facilitated pack and a resource file. | Care home staff. | NR |
| Wickson-Griffiths et al, 2015 (72)  Canada  Semi structured individual /focus group interviews | Comfort Care Rounds (CCRs) Strategy. | **External facilitation:** A palliative care consultant, comprehensive advanced palliative care education trained nurse, and other CCRs leaders (e.g. nurse managers) were responsible for chairing or co-chairing CCRs with interprofessional staff and palliative care volunteers. Responsibilities included developing an agenda, promoting and advertising CCRs, facilitating discussion, providing education, and disseminating key messages to staff not in attendance.  **Internal facilitation:** CCRs leaders (e.g. nurse managers) were responsible for chairing or co-chairing CCRs. | CCRs were scheduled on a monthly basis, for 30 minutes to 1 hour. | All members of the interprofessional  team and palliative care volunteers. | Two palliative care physicians from under a medical director contract provided consultation. |
| Wils et al, 2017 (73)  Belgium  Evaluation | Conceptual Framework for Implementation of ACP (model of care goals). | **External facilitation:** The educational training sessions were given by one of the researchers who had been trained in ACP.  **Internal facilitation:** NR | Two educational training sessions and four debriefing sessions, lasting two hours each. A number of conversations with the residents were filmed and discussed in the intervention group. | Registered nurses. | NR |

**Abbreviations**

ACP - Advance care planning
CARES - Caring About Residents’ Experience and Symptoms
CCRs - Comfort Care Rounds
DNACPR - Do not attempt cardiopulmonary resuscitation
DNAR - Do not attempt resuscitation
ESAS - Edmonton Symptom Assessment
GP - General practitioner
CP - Integrated care pathway
LCP - Liverpool care pathway
LTCF - Long term care facility
MMRI-R - Minimum Dataset Mortality Risk Index – Risk
MOST - Medical orders for scope of treatment
PCPC - Palliative care planning coordinator
VOICES - Views of Informal Carers – Evaluation of Service

References

1. Aasmul I, Husebo BS, Flo E. Description of an advance care planning intervention in nursing homes: outcomes of the process evaluation. BMC Geriatr. 2018;18:11.

2. Agar M, Luckett T, Luscombe G, Phillips J, Beattie E, Pond D, et al. Effects of facilitated family case conferencing for advanced dementia: A cluster randomised clinical trial. PloS one. 2017;12(8):e0181020.

3. Luckett T, Chenoweth L, Phillips J, Brooks D, Cook J, Mitchell G, et al. A facilitated approach to family case conferencing for people with advanced dementia living in nursing homes: perceptions of palliative care planning coordinators and other health professionals in the IDEAL study. International Psychogeriatrics. 2017;29(10):1713-22.

4. Amador S, Goodman C, Mathie E, Nicholson C. Evaluation of an Organisational Intervention to Promote Integrated Working between Health Services and Care Homes in the Delivery of End-of-Life Care for People with Dementia: Understanding the Change Process Using a Social Identity Approach. International Journal of Integrated Care. 2016;16(2):14.

5. Ampe S, Sevenants A, Smets T, Declercq A, Van Audenhove C. Advance care planning for nursing home residents with dementia: Influence of 'we DECide' on policy and practice. Patient Education and Counseling. 2017;100(1):139-46.

6. Andrews S, McInerney F, Robinson A. Realizing a palliative approach in dementia care: strategies to facilitate aged care staff engagement in evidence-based practice. International Psychogeriatrics. 2009;21:S64-S8.

7. Arcand M, Monette J, Monette M, Sourial N, Fournier L, Gore B, et al. Educating Nursing Home Staff About the Progression of Dementia and the Comfort Care Option: Impact on Family Satisfaction with End-of-Life Care. Journal of the American Medical Directors Association. 2009;10(1):50-5.

8. Badger F, Thomas K, Clifford C. Raising standards for elderly people dying in care homes. European Journal of Palliative Care. 2007;14(6):238-41.

9. Badger F, Clifford C, Hewison A, Thomas K. An evaluation of the implementation of a programme to improve end-of-life care in nursing homes. Palliat Med. 2009;23(6):502-11.

10. Badger F, Plumridge G, Hewison A, Shaw KL, Thomas K, Clifford C. An evaluation of the impact of the Gold Standards Framework on collaboration in end-of-life care in nursing homes. A qualitative and quantitative evaluation. International Journal of Nursing Studies. 2012;49(5):586-95.

11. Beck I, Törnquist A, Edberg AK. Nurse assistants' experience of an intervention focused on a palliative care approach for older people in residential care. International Journal of Older People Nursing. 2012;9(2):140-50.

12. Beck I, Jakobsson U, Edberg AK. Applying a palliative care approach in residential care: Effects on nurse assistants’ work situation. Palliative and Supportive Care 2015;13:543–53.

13. Blackford J, Strickland E, Morris B. Advance care planning in residential aged care facilities. Contemporary Nurse. 2007;27(1):141-51.

14. Booth M, Nash S, Banks C, Springett A. Three approaches to delivering end-of-life education to care homes in a region of south east England. International Journal of Palliative Nursing. 2014;20(1):27-35.

15. Brajtman S, Wright D, Hall P, Bush SH, Bekele E. Toward better care of delirious patients at the end of life: A pilot study of an interprofessional educational intervention. Journal of Interprofessional Care. 2012;26(5):422-5.

16. Brannstrom M, Furst CJ, Tishelman C, Petzold M, Lindqvist O. Effectiveness of the Liverpool care pathway for the dying in residential care homes: An exploratory, controlled before-and-after study. Palliat Med. 2016;30(1):54-63.

17. Campion C, Kassaye A, Sutherland S, Carruthers M, Riley J, Wood J, et al. Improving end-of-life care in nursing homes: an innovative model of education and training. European Journal of Palliative Care. 2016;23(5):222-6.

18. Chapman M, Johnston N, Lovell C, Forbat L, Liu WM. Avoiding costly hospitalisation at end of life: findings from a specialist palliative care pilot in residential care for older adults. BMJ Supportive & Palliative Care. 2018;8(1):102-9.

19. Chisholm L, Zimmerman S, Rosemond C, McConnell E, Weiner B, Lin F-C, et al. Nursing home staff perspectives on adoption of an innovation ingoals of care communication. Geriatric Nursing 2017;39 (2):157-61.

20. Hanson LC, Song MK, Zimmerman S, Gilliam R, Rosemond C, Chisholm L, et al. Fidelity to a behavioral intervention to improve goals of care decisions for nursing home residents with advanced dementia. Clinical Trials. 2016;13(6):599-604.

21. Hanson LC, Zimmerman S, Song MK, Lin FC, Rosemond C, Carey TS, et al. Effect of the Goals of Care Intervention for Advanced Dementia A Randomized Clinical Trial. JAMA Internal Medicine. 2017;177(1):24-31.

22. Cornally N, McGlade C, Weathers E, Daly E, Fitzgerald C, O'Caoimh R, et al. Evaluating the systematic implementation of the 'Let Me Decide' advance care planning programme in long term care through focus groups: staff perspectives. BMC Palliative Care. 2015;14.

23. Cox A, Arber A, Bailey F, Dargan S, Gannon C, Lisk R, et al. Developing, implementing and evaluating an end of life care intervention. Nursing Older People. 2017;29(1):27-35.

24. Cronfalk BS, Ternestedt BM, Larsson LLF, Henriksen E, Norberg A, Osterlind J. Utilization of palliative care principles in nursing home care: Educational interventions. Palliative & Supportive Care. 2015;13(6):1745-53.

25. Farrington CJT. Blended e-learning and end of life care in nursing homes: a small-scale mixed-methods case study. BMC Palliative Care. 2014;13.

26. Fernandes G. Implementation of best practice in advance care planning in an 'ageing in place' aged care facility. International Journal of Evidence-Based Healthcare. 2008;6(2):270-6.

27. Finucane AM, Stevenson B, Moyes R, Oxenham D, Murray SA. Improving end-of-life care in nursing homes: Implementation and evaluation of an intervention to sustain quality of care. Palliat Med. 2013;27(8):772-8.

28. Frey R, Boyd M, Robinson J, Foster S, Gott M. The Supportive Hospice and Aged Residential Exchange (SHARE) programme in New Zealand. Nurse Education in Practice. 2017;25:80-8.

29. Garden G, Green S, Pieniak S, Gladman J. The Bromhead Care Home Service: the impact of a service for care home residents with dementia on hospital admission and dying in preferred place of care. Clinical Medicine. 2016;16(2):114-8.

30. Giuffrida J. Palliative Care in Your Nursing Home: Program Development and Innovation in Transitional Care. Journal of Social Work in End-of-Life & Palliative Care. 2015;11(2):167-77.

31. Hall S, Goddard C, Opio D, Speck P, Higginson IJ. Feasibility, acceptability and potential effectiveness of Dignity Therapy for older people in care homes: A phase II randomized controlled trial of a brief palliative care psychotherapy. Palliat Med. 2012;26(5):703-12.

32. Hasson F, Kernohan WG, Waldron M, Whittaker E, McLaughlin D. The palliative care link nurse role in nursing homes: barriers and facilitators. Journal of advanced nursing. 2008;64(3):233-42.

33. Hewison A, Badger F, Clifford C, Thomas K. Delivering 'Gold Standards' in end-of-life care in care homes: a question of teamwork? Journal of Clinical Nursing. 2009;18(12):1756-65.

34. Hickman SE, Unroe KT, Ersek MT, Buente B, Nazir A, Sachs GA. An Interim Analysis of an Advance Care Planning Intervention in the Nursing Home Setting. Journal of the American Geriatrics Society. 2016;64(11):2385-92.

35. Ho AHY, Dai AAN, Lam SH, Wong SWP, Tsui ALM, Tang CS, et al. Development and Pilot Evaluation of a Novel Dignity Conserving End-of-Life (EoL) Care Model for Nursing Homes in Chinese Societies. Gerontologist. 2016;56(3):578-89.

36. Ho AHY, Luk JKH, Chan FHW, Ng WC, Kwok CKK, Yuen JHL, et al. Dignified Palliative Long-Term Care: An Interpretive Systemic Framework of End-of-Life Integrated Care Pathway for Terminally Ill Chinese Older Adults. American Journal of Hospice & Palliative Medicine. 2016;33(5):439-47.

37. Hockley J, Kinley J. A practice development initiative supporting care home staff deliver high quality end-of-life care. International Journal of Palliative Nursing. 2016;22(10):474-81.

38. Hockley J, Watson J, Oxenham D, Murray SA. The integrated implementation of two end-of-life care tools in nursing care homes in the UK: an in-depth evaluation. Palliat Med. 2010;24(8):828-38.

39. Watson J, Hockley J, Murray S. Evaluating effectiveness of the GSFCH and LCP in Care Homes. End of Life Care Journal. 2010;4(3).

40. Horey DE, Street AF, Sands AF. Acceptability and feasibility of end-of-life care pathways in Australian residential aged care facilities. Medical Journal of Australia. 2012;197(2):106-9.

41. In der Schmitten J, Lex K, Mellert C, Rotharmel S, Wegscheider K, Marckmann G. Implementing an advance care planning program in German nursing homes: results of an inter-regionally controlled intervention trial. Deutsches Arzteblatt International. 2014;111(4):50-7.

42. Kataoka-Yahiro MR, McFarlane S, Koijane J, Li D. Culturally Competent Palliative and Hospice Care Training for Ethnically Diverse Staff in Long-Term Care Facilities. American Journal of Hospice & Palliative Medicine. 2017;34(4):335-46.

43. Kinley J, Stone L, Dewey M, Levy J, Stewart R, McCrone P, et al. The effect of using high facilitation when implementing the Gold Standards Framework in Care Homes programme: A cluster randomised controlled trial. Palliat Med. 2014;28(9):1099-109.

44. Kinley J, Preston N, Froggatt K. Facilitation of an end-of-life care programme into practice within UK nursing care homes: A mixed-methods study. International Journal of Nursing Studies. 2018;82:1-10.

45. Kinley J, Stone L, Butt A, Kenyon B, Lopes NS. Developing, implementing and sustaining an end-of-life care programme in residential care homes. International Journal of Palliative Nursing. 2017;23(4):186-93.

46. Knight G, Jordan C, Lewis M. Improving end-of-life care in the care home sector. Practice Development in Health Care. 2008;7(4):189-97.

47. Kortes-Miller K, Habjan S, Kelley ML, Fortier M. Development of a palliative care education program in rural long-term care facilities. Journal of Palliative Care. 2007;23(3):154-62.

48. Kortes-Miller K, Jones-Bonofiglio K, Hendrickson S, Kelley ML. Dying With Carolyn: Using Simulation to Improve Communication Skills of Unregulated Care Providers Working in Long-Term Care. Journal of Applied Gerontology. 2015;35(12):1259-78.

49. Kuhn DR, Forrest JM. Palliative Care for Advanced Dementia: A Pilot Project in 2 Nursing Homes. American Journal of Alzheimers Disease and Other Dementias. 2012;27(1):33-40.

50. Lansdell J, Mahoney M. An end of life care training programme from hospice staff. Nursing and Residential Care. 2011;13(9):438-40.

51. Livingston G, Lewis-Holmes E, Pitfield C, Manela M, Chan D, Constant E, et al. Improving the end-of-life for people with dementia living in a care home: an intervention study. International Psychogeriatrics. 2013;25(11):1849-58.

52. Lyon C. Advance care planning for residents in aged care facilities: What is best practice and how can evidence-based guidelines be implemented? International Journal of Evidence-Based Healthcare. 2007;5(4):450-7.

53. Magee M, McCorkell G, Guille S, Coates V. Feasibility of the Namaste Care Programme to enhance care for those with advanced dementia. International Journal of Palliative Nursing. 2017;23(8):368-76.

54. Mayrhofer A, Goodman C, Smeeton N, Handley M, Amador S, Davies S. The feasibility of a train-the-trainer approach to end of life care training in care homes: an evaluation. BMC Palliative Care. 2016;15.

55. McGlade C, Daly E, McCarthy J, Cornally N, Weathers E, O'Caoimh R, et al. Challenges in implementing an advance care planning programme in long-term care. Nursing Ethics. 2017;24(1):87-99.

56. Saini G, Sampson EL, Davis S, Kupeli N, Harrington J, Leavey G, et al. An ethnographic study of strategies to support discussions with family members on end-of-life care for people with advanced dementia in nursing homes. BMC Palliative Care. 2016;15.

57. Moore KJ, Candy B, Davis S, Gola A, Harrington J, Kupeli N, et al. Implementing the compassion intervention, a model for integrated care for people with advanced dementia towards the end of life in nursing homes: a naturalistic feasibility study. BMJ Open. 2017;7(6).

58. Morris DA, Galicia-Castillo M. Caring About Residents’ Experiences and Symptoms (CARES) Program: A Model of Palliative Care Consultation in the Nursing Home. American Journal of Hospice & Palliative Medicine. 2017;34(5):466-9.

59. Nilsen P, Wallerstedt B, Behm L, Ahlstrom G. Towards evidence-based palliative care in nursing homes in Sweden: a qualitative study informed by the organizational readiness to change theory. Implementation Science. 2018;13:12.

60. O'Brien M, Kirton J, Knighting K, Roe B, Jack B. Improving end of life care in care homes; an evaluation of the six steps to success programme. BMC Palliative Care. 2016;15.

61. Oliver DP, Bickel-Swenson D, Zweig S, Kruse R, Mehr D. Experience with implementation of a quality improvement project for the care of nursing home residents. Journal of Nursing Care Quality. 2009;24(2):100-4.

62. Raunker M, Timm H. Development of palliative care in nursing homes: evaluation of a Danish project. International Journal of Palliative Nursing. 2010;16(12):613-20.

63. Stacpoole M, Hockley J, Thompsell A, Simard J, Volicer L. The Namaste Care programme can reduce behavioural symptoms in care home residents with advanced dementia. International Journal of Geriatric Psychiatry. 2015;30(7):702-9.

64. Stacpoole M, Hockley J, Thompsell A, Simard J, Volicer L. Implementing the Namaste Care Program for residents with advanced dementia: exploring the perceptions of families and staff in UK care homes. Ann. 2017;6(4):327-39.

65. Taylor AJ, Randall C. Process mapping: enhancing the implementation of the Liverpool Care Pathway. International Journal of Palliative Nursing. 2007;13(4):163-7.

66. Temkin-Greener H, Ladwig S, Ye ZQ, Norton SA, Mukamel DB. Improving palliative care through teamwork (IMPACTT) in nursing homes: Study design and baseline findings. Contemporary Clinical Trials. 2017;56:1-8.

67. Temkin-Greener H, Mukamel DB, Ladd H, Ladwig S, Caprio TV, Norton SA, et al. Impact of Nursing Home Palliative Care Teams on End-of-Life Outcomes: A Randomized Controlled Trial. Medical Care. 2017;56(1):11-8.

68. Unroe KT, Nazir A, Holtz LR, Maurer H, Miller E, Hickman SE, et al. The Optimizing Patient Transfers, Impacting Medical Quality, and Improving Symptoms: Transforming Institutional Care Approach: Preliminary Data from the Implementation of a Centers for Medicare and Medicaid Services Nursing Facility Demonstration Project. Journal of the American Geriatrics Society. 2015;63(1):165-9.

69. Verreault R, Arcand M, Misson L, Durand PJ, Kroger E, Aubin M, et al. Quasi-experimental evaluation of a multifaceted intervention to improve quality of end-of-life care and quality of dying for patients with advanced dementia in long-term care institutions. Palliat Med. 2018;32(3):613-21.

70. Vis JA, Ramsbottom K, Marcella J, McAnulty J, Kelley ML, Kortes-Miller K, et al. Developing and Implementing Peer-Led Intervention to Support Staff in Long-Term Care Homes Manage Grief. Sage Open. 2016;6(3).

71. Waldron M, Hasson F, Kernohan WG, Whittaker E, McClaughlin D. Evaluating education in palliative care with link nurses in nursing homes. British Journal of Nursing. 2008;17(17):1078-83.

72. Wickson-Griffiths A, Kaasalainen S, Brazil K, McAiney C, Crawshaw D, Turner M, et al. Comfort Care Rounds A Staff Capacity-Building Initiative in Long-Term Care Homes. Journal of Gerontological Nursing. 2015;41(1):42-9.

73. Wils M, Verbakel J, Lisaerde J. Improving advance care planning in patients with dementia: the effect of training nurses to engage in ACP-related conversations. Journal of Clinical Gerontology & Geriatrics. 2017;8(1):17-20.
